# Combined impact of smoking, alcohol and social inequalities on premature mortality in urban India

**DOI:** 10.1038/s44360-026-00148-4

**Published:** 2026-06-19

**Authors:** Vendhan Gajalakshmi, Fiona Bragg, Ben Lacey, Vendhan Kanimozhi, Manigandan Chandrasekaran, Ramu Ashok Kumar, Paul Sherliker, Richard Peto, Sarah Lewington

**Affiliations:** 1 Epidemiological Research Centre, Chennai, India; 2 Nuffield Department of Population Health, University of Oxford, Oxford, UK; 3 Health Data Research UK Oxford, University of Oxford, Oxford, UK; 4 UK Biobank, Stockport, UK; 5 Liverpool Women’s Hospital NHS Foundation Trust, Liverpool, UK

**Keywords:** Risk factors, Epidemiology, Diseases

## Abstract

Associations with mortality of social inequalities, smoking and drinking differ widely between populations. Here we recruited 500,810 adults aged ≥35 years from Chennai city during 1998–2001 in South India and followed to 2020 for verbal-autopsy-assessed cause-specific mortality. Among those initially without chronic disease, multivariable-adjusted Cox regression (censored at age 70 years) related initial characteristics to mortality rates. Even among non-smoking non-drinkers, those with no schooling had almost three times the mortality rate ratio (RR) of those with >11 years education (men, RR of 2.76, 95% confidence interval (CI) 2.58–2.95; women, RR of 2.93, 95% CI 2.69–3.19), with substantial mortality excesses from each major disease category. These social inequalities were further exacerbated by much higher smoking and drinking prevalences among less educated men. Adjusted for education, male smoking (versus not, RR of 1.26, 95% CI 1.22–1.29) and drinking (versus not, RR of 1.52, 95% CI 1.48–1.56) were independently associated with risk and together almost doubled male mortality (both versus neither, RR of 1.89, 95% CI 1.84–1.94). Few women (<0.1%) smoked or drank.

## Abstract (web summary)

A 20-year prospective cohort study of 458,609 adults in Chennai shows that educational disadvantage is associated with premature mortality even among non-smokers and non-drinkers, and in men, smoking and alcohol use amplify these social inequalities, with combined use nearly doubling mortality risk before age 70.

## Main

In many populations, substantial health inequalities persist from childhood into adult life, with lower education or socioeconomic status (SES) associated with a higher burden of alcohol drinking and smoking^1–4^ and with higher adult mortality rates^5^ over and above any effects of smoking or drinking. Most studies of the separate and combined associations of social inequalities, smoking and drinking with mortality have been in Western populations^6–12^; the findings in other populations could well be very different.

We report, to our knowledge, the largest prospective study in India—involving 500,000 urban men and women aged ≥35 years in Chennai city—of the independent and joint associations of education, adult SES, smoking and alcohol drinking with mortality before the age of 70 years.

## Results

The main analyses excluded 33,323 recruits aged ≥70 years plus 8,878 with chronic disease, leaving 458,609 (Supplementary Table 2). The baseline characteristics varied with years of education (Table 1) and age (Supplementary Table 3). The mean age was 46 years (s.d. 9), indicating mid-century birth. Particularly for women, those born more recently had more education than those born earlier. Few women (<0.1%) ever smoked or drank. Among men, the lack of education was strongly associated with low SES and with the prevalence of smoking or drinking (65% (15,145 out of 23,385) with no schooling ever smoked or drank versus 21% (8,828 out of 42,688) with >11 years of education).

**Table 1:** Baseline characteristics at ages 35–69 years, by years of education and sex

	Years of education				Overall
	None	1–7	8–11	>11	
Men, *N*	23,385	78,595	98,424	42,688	243,092
Age (years)	47.8 (10.0)	46.3 (9.6)	45.4 (8.9)	45.1 (8.5)	45.9 (9.2)
Current tobacco smoker	12,396 (53%)	36,047 (46%)	30,382 (31%)	6,782 (16%)	85,607 (35%)
Total no. of cigarettes or bidi per day	13 (8)	12 (7)	11 (6)	10 (6)	11 (7)
Only cigarettes					
* N* (% of current)	5,399 (44%)	22,171 (62%)	24,342 (80%)	6,360 (94%)	58,272 (68%)
Cigarettes per day	10 (5)	10 (5)	10 (5)	9 (5)	10 (5)
Only bidis					
* N* (% of current)	5,781 (47%)	11,040 (31%)	4,646 (15%)	300 (4%)	21,767 (25%)
Bidi/day	15 (8)	15 (8)	14 (7)	13 (7)	14 (8)
Remaining current smokers					
*N* (% of current)	1,216 (10%)	2,836 (8%)	1,394 (5%)	122 (2%)	5,568 (7%)
Total of cigarettes or bidi per day	19 (11)	19 (11)	18 (11)	16 (13)	19 (11)
Ex-smokers, *N* (%)	507 (2%)	1,886 (2%)	1,599 (2%)	572 (1%)	4,564 (2%)
Ever tobacco smoker	12,903 (55%)	37,933 (48%)	31,981 (33%)	7,354 (17%)	90,171 (37%)
Total of cigarettes or bidi per day when last smoked	13 (8)	12 (7)	11 (6)	10 (6)	11 (7)
Age at starting	21.2 (4.2)	21.7 (4.2)	22.3 (4.1)	23.0 (4.1)	21.9 (4.2)
Current alcohol drinker	10,506 (45%)	27,418 (35%)	21,682 (22%)	4,206 (10%)	63,812 (26%)
Alcohol per week (g)^a^	211 (431)	193 (440)	172 (429)	160 (497)	187 (439)
Ever drank alcohol	10,951 (47%)	28,833 (37%)	22,784 (23%)	4,482 (11%)	67,050 (28%)
Alcohol per week (g) when last drank	215 (458)	195 (446)	174 (429)	164 (566)	189 (452)
Ex-alcohol drinker, *N* (%)	445 (2%)	1,415 (2%)	1,102 (1%)	276 (1%)	3,238 (1%)
Ever smoked or drank	15,145 (65%)	44,001 (56%)	38,176 (39%)	8,828 (21%)	106,150 (44%)
Ever smoked, drank or chewed	16,130 (69%)	46,326 (59%)	40,679 (41%)	9,581 (22%)	112,716 (46%)
Vegetarian	184 (1%)	1,014 (1%)	4,329 (4%)	4,555 (11%)	10,082 (4%)
Current quid-chewing^b^	2,689 (12%)	5,582 (7%)	4,681 (5%)	1,145 (3%)	14,097 (6%)
Lowest SES group	11,694 (50%)	29,337 (37%)	20,131 (21%)	2,579 (6%)	63,741 (26%)
BMI (kg m^−^^2^)	21.4 (3.2)	22.1 (3.3)	22.9 (3.2)	23.6 (3.2)	22.6 (3.3)
BMI <20	7,017 (30%)	17,857 (23%)	14,895 (15%)	3,646 (9%)	43,415 (18%)
Self-reported diabetes	558 (2%)	3,257 (4%)	5,094 (5%)	2,513 (6%)	11,422 (5%)
SBP	127 (15)	127 (14)	128 (14)	128 (13)	128 (14)
DBP	82 (9)	83 (9)	84 (9)	84 (8)	83 (9)
Hypertension	6,206 (28%)	22,022 (29%)	29,371 (31%)	12,944 (33%)	70,543 (31%)
Treated, *N* (% of hypertension)	429 (7%)	2,276 (10%)	3,688 (13%)	1,923 (15%)	8,316 (12%)
Women, *N*	62,907	81,616	57,239	13,755	215,517
Age (years)	49.7 (9.8)	46.5 (9.3)	44.5 (8.3)	43.4 (7.5)	46.7 (9.4)
Ever tobacco smoker	52 (0.1%)	38 (0.0%)	7 (0.0%)	3 (0.0%)	100 (0.0%)
Ever drank alcohol	62 (0.1%)	31 (0.0%)	17 (0.0%)	2 (0.0%)	112 (0.1%)
Ever smoked or drank	104 (0.2%)	60 (0.1%)	23 (0.0%)	4 (0.0%)	191 (0.1%)
Vegetarian	3,204 (5%)	5,982 (7%)	7,279 (13%)	2,482 (18%)	18,947 (9%)
Current quid-chewing^b^	13,372 (21%)	5,626 (7%)	1,568 (3%)	184 (1%)	20,750 (10%)
Lowest SES group	27,132 (43%)	22,127 (27%)	7,816 (14%)	619 (5%)	57,694 (27%)
BMI (kg m^−^^2^)	23.1 (4.4)	24.2 (4.5)	24.6 (4.3)	24.8 (4.0)	24.0 (4.4)
BMI <20	13,136 (21%)	11,657 (14%)	6,329 (11%)	1,016 (7%)	32,138 (15%)
Self-reported diabetes	3,296 (5%)	4,729 (6%)	2,683 (5%)	502 (4%)	11,210 (5%)
SBP	128 (18)	127 (18)	126 (16)	125 (15)	127 (17)
DBP	83 (11)	83 (10)	82 (10)	82 (9)	83 (10)
Hypertension	19,295 (34%)	24,291 (33%)	14,767 (30%)	2,936 (26%)	61,289 (32%)
Treated, *N* (% of hypertension)	3,057 (16%)	4,689 (19%)	2,794 (19%)	516 (18%)	11,056 (18%)

Values are % (and number) or mean (s.d.), as appropriate.

For systolic blood pressure (SBP), diastolic blood pressure (DBP), some participants did not contribute values for one or more of these variables.

Hypertension, SBP ≥140 or DBP ≥90 mm Hg or treatment for hypertension (as a surrogate for diagnosis, as this was not directly reported).

^a^Past/current intake, assuming alcohol by volume: spirits 40%, wine 12%, beer 4%, toddy 5%.

^b^Any quid-chewing: tobacco, non-tobacco or combination, whether regular or occasional.

Education and SES at baseline and at resurvey about 14 years later, by sex.

SES score is a simple summation of values from each SES element of the questionnaire. Housing type, hut, 1; portion, 2; flat, 3; individual, 4; rooms: point per room (maximum 4); separate bathroom, no, 0; yes, 1; fuel used for cooking, wood, 1; kerosene, 2; gas, 3; vehicle, none, 0; cycle, 1; two-wheeler, 2; other, 3; car, 4; refrigerator, no, 0; yes, 1; house owner, tenant, 0; owner, 1; TV: no, 0; black and white, 1; colour, 2; grinder, no, 0; yes, 1. The SES cut points are quartiles of the baseline score.

The 7,127 men and 3,006 women resurveyed in 2013–2014, about 14 years after baseline, had, at baseline, had fairly typical patterns of education, SES, smoking and drinking (Table 1 and Supplementary Tables 4 and 5). Self-reported educational level (four categories) was highly reproducible and unchanged in 91% (9,192 out of 10,133) of those resurveyed, but the study-assessed SES score was poorly reproducible, partly owing to increasing household assets (Supplementary Table 4). Among men, at resurvey, 7.5% (182 out of 2,410) of those who reported current smoking at baseline had quit, and 7.8% (161 out of 2,066) of those who reported current drinking at baseline had quit. Hence, 97% (6,900 out of 7,127) reported the same smoking status at baseline and resurvey and 96% (6,870 out of 7,127) reported the same drinking status at baseline and resurvey; smoking (ever/never) and drinking (ever/never) were both unchanged in >99%, suggesting high reliability (Supplementary Table 5). Although the amounts reportedly smoked and drunk by individuals fluctuated between baseline and resurvey, the average number of cigarettes/bidi smoked among men who smoked reduced only slightly between baseline and resurvey (Supplementary Table 6), whereas the mean consumption of alcohol among men who drank increased slightly (Supplementary Table 7). The usual consumption among all study participants in the baseline-assigned categories of smoking and drinking is estimated as the mean consumption at resurvey in those baseline-assigned categories.

**Fig. 1:**
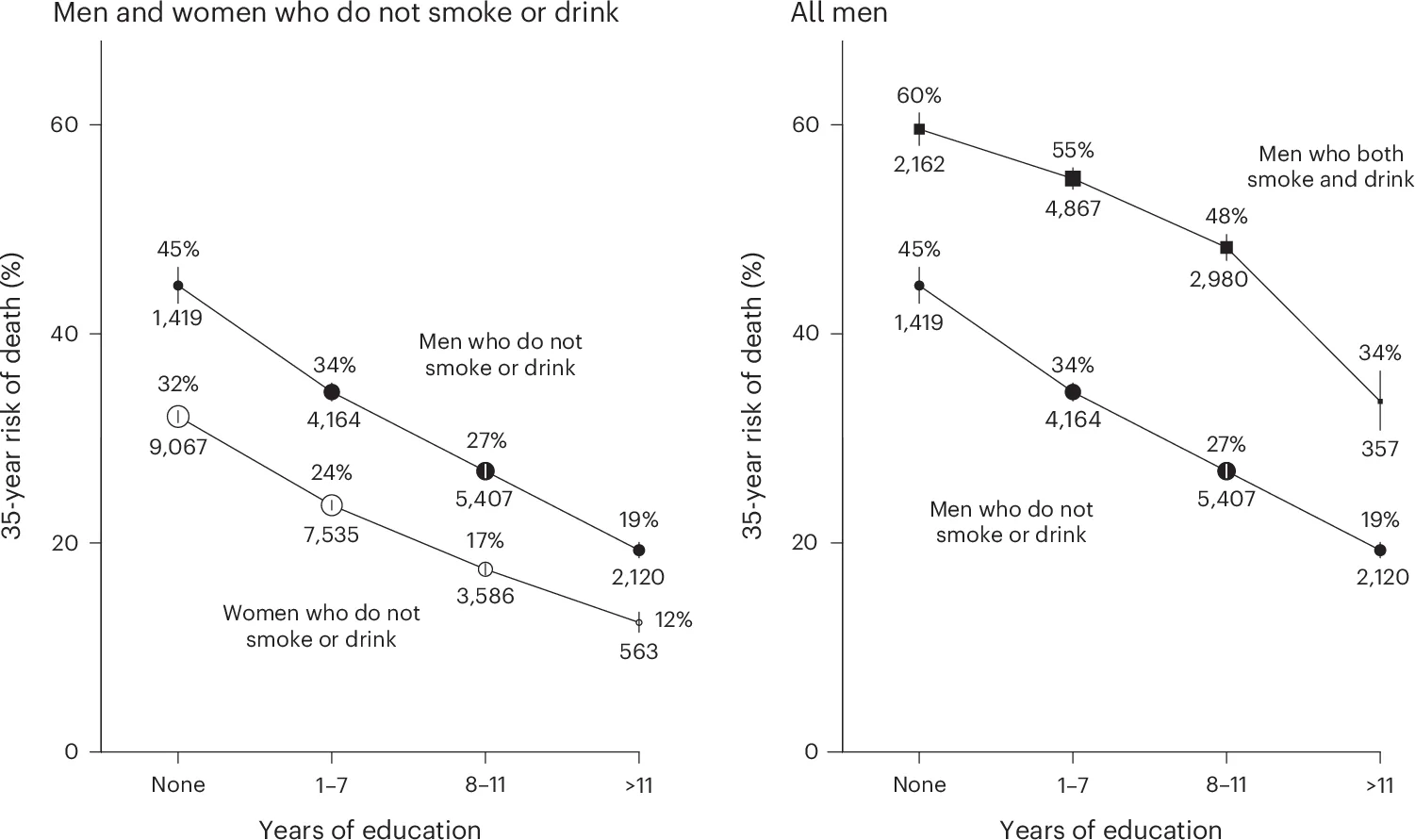
Associations of education, sex, smoking and drinking with risk of death at ages 35–69 years. The probability that a 35-year-old would die before age 70 years was estimated by combining age-standardized mortality RRs with the Registrar-General of India’s age–sex-specific Chennai mortality rates. The 35-year risk is shown above or beside each point and the number of events below. For each group, the 95% CI reflects the variance of the log risk in that group, and the area of the plotted point is inversely proportional to this variance.

**Extended Data Fig. 1:**
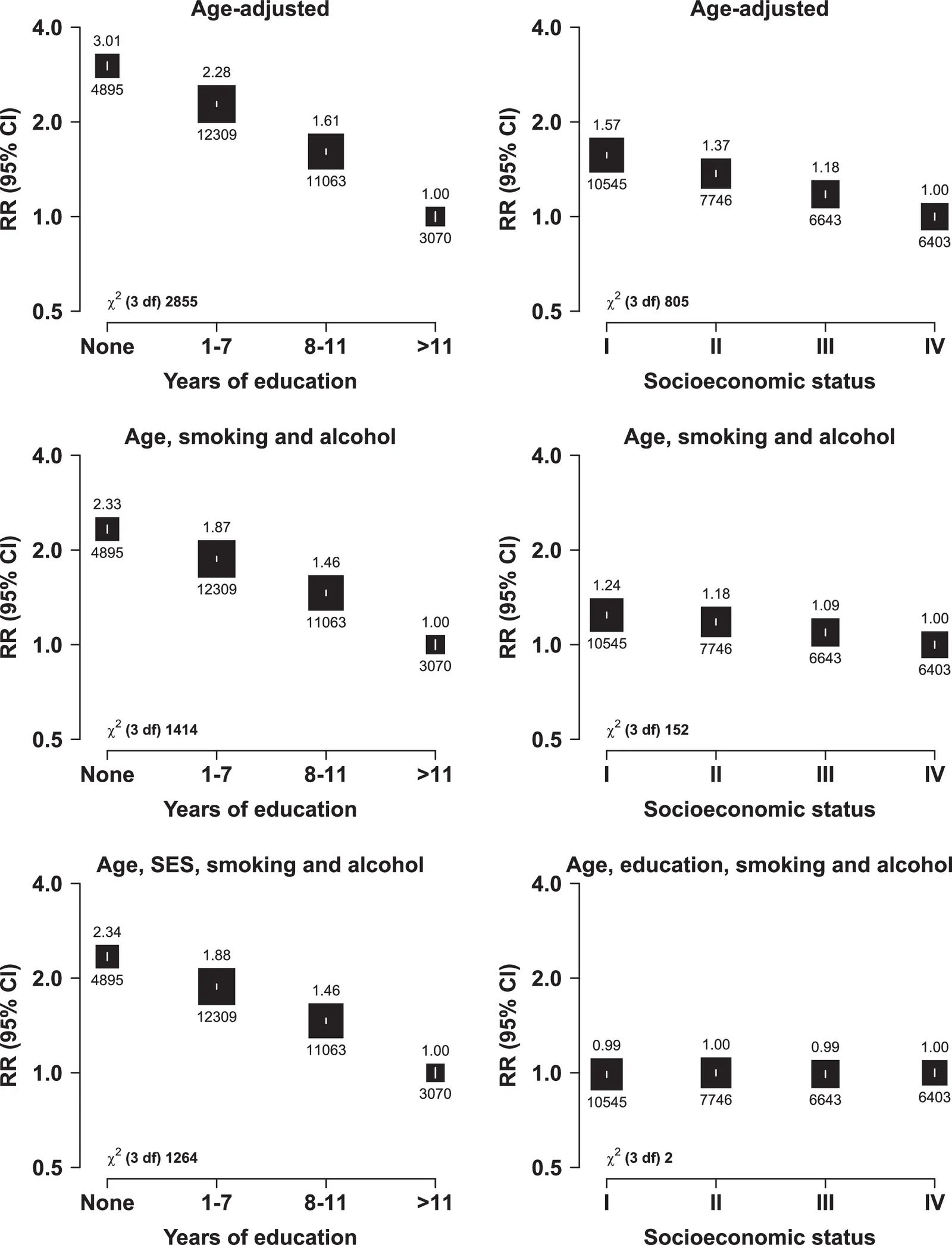
Associations of education and of SES with all-cause mortality rate ratios among men at ages 35-69 years before and after adjustment for smoking, alcohol and each other. Mortality rate ratios (RR) are standardised for age. Men with >11 years education had RR = 1. The RR is shown above each square and the number of events below. For each group, the 95% CI reflects the variance of the log risk of that group, and the area of the plotted point is inversely proportional to this variance.

**Extended Data Fig. 2:**
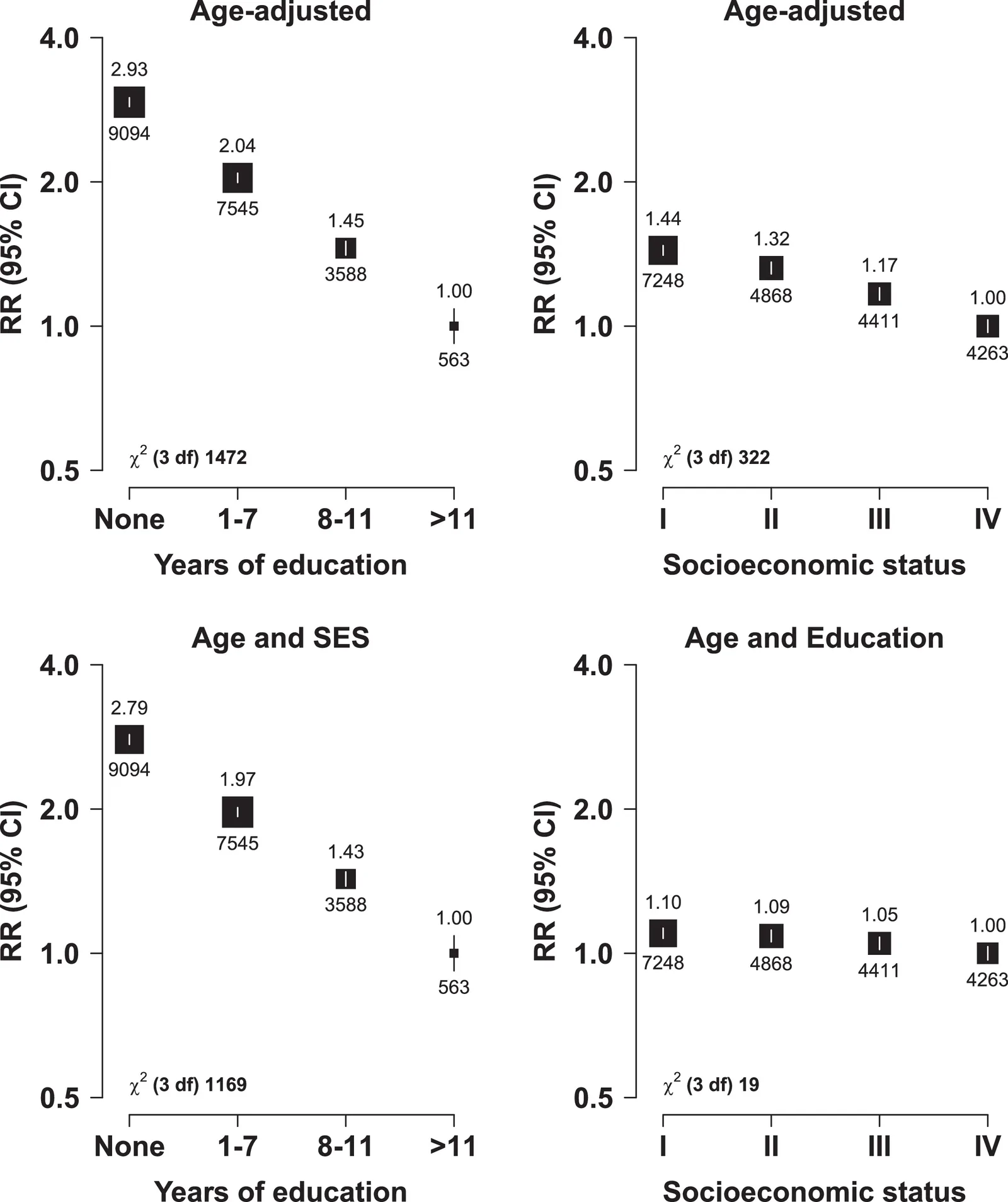
Associations of education and of SES with all-cause mortality rate ratios among women at ages 35-69 years before and after adjustment for smoking, alcohol and each other. Mortality rate ratios (RR) are standardised for age. Women with >11 years education had RR = 1. The RR is shown above each square and the number of events below. For each group, the 95% CI reflects the variance of the log risk of that group, and the area of the plotted point is inversely proportional to this variance. Additional adjustment for smoking and drinking among women is not presented since <0.2% of women smoke or drink in any education group.

During 20-year follow-up (16.4 ± 5.4 (mean ± s.d.) person-years at risk), there were 52,127 deaths at ages 35–69 years (31,337 men and 20,790 women), of which 47,436 (91%) were identified by active follow-up and 4,691 (9%) were identified through passive follow-up alone. Among men and women who never smoked or drank, education was strongly inversely associated with mortality (Fig. 1a). On average, those with no schooling had about three times the overall mortality rate at ages 35–69 years of those with tertiary education (men, mortality RR of 2.76, 95% CI 2.58–2.95; women, RR of 2.93, 95% CI 2.69–3.19) and about twice the overall mortality rate of those with 8–11 years of education. As SES was strongly correlated with education it was also inversely associated with mortality, although not as strongly as was education. In multivariate analyses relating all-cause mortality jointly to baseline SES score (quartiles) and years of education (four categories) SES was of little independent relevance, but the relevance of education remained unchanged (Extended Data Figs. 1 and 2).

Within each educational level, among those who never smoked or drank, the mortality rate in men was about 1.5 times that in women. Few women smoked or drank, but at each educational level, men who smoked and drank had substantially higher mortality than men who did neither (Fig. 1b). Smoking and drinking were both strongly inversely associated with education, and subsequent analyses of associations between educational level and mortality omit (unless stated otherwise) participants who ever smoked or drank.

**Fig. 2:**
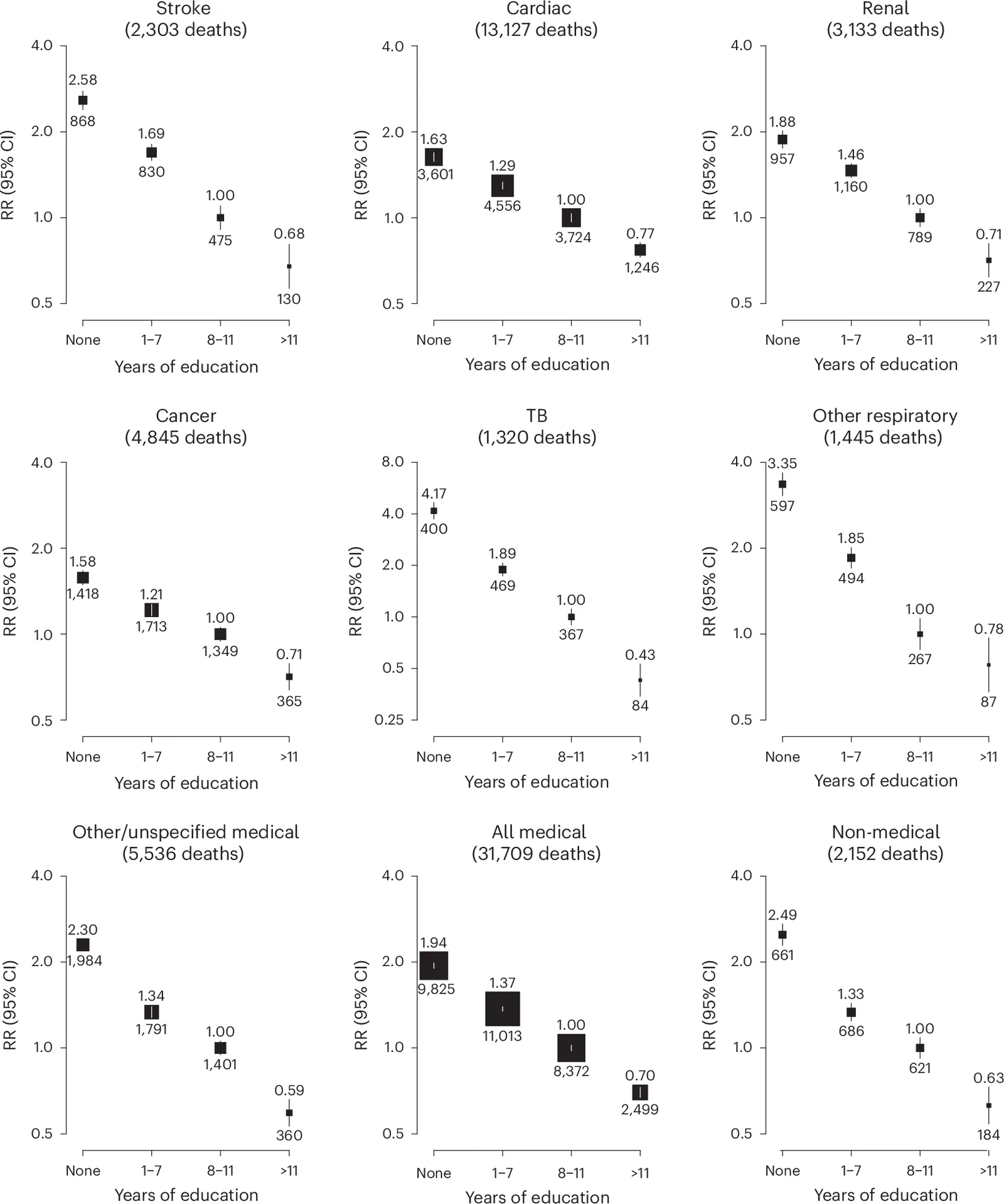
Associations of education with cause-specific mortality RRs at ages 35–69 years among adults who do not smoke or drink. Mortality RRs are standardized for age and sex. Adults with 8–11 years education had an RR of 1; those with >11 years education had an RR <1 for each cause. The RR is shown above each square and the number of events below. For each group, the 95% CI reflects the variance of the log risk, and the area of the plotted point is inversely proportional to this variance. The TB vertical scale is compressed, owing to the 20-fold mortality range. ‘Other’ includes all known medical causes except those categorized above. ‘Unspecified’ are non-specific causes.

**Extended Data Fig. 3:**
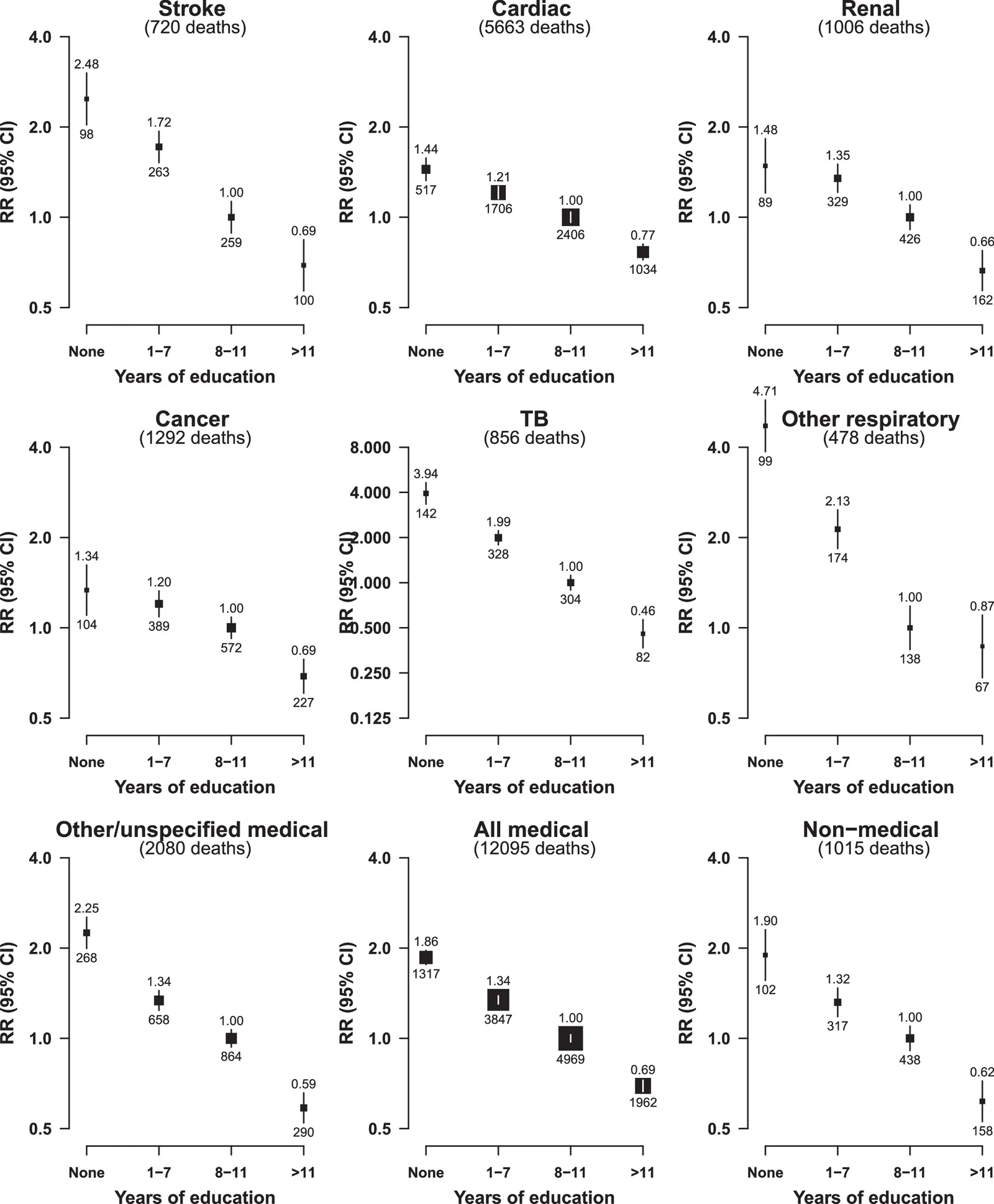
Associations of education with cause−specific mortality rate ratios at ages 35 − 69 years among men who do not smoke or drink. Mortality rate ratios (RR) are standardised for age. Men with 8-11 years education had RR = 1; those with >11 years education had RR < 1 for each cause. The RR is shown above each square and the number of events below. For each group, the 95% CI reflects the variance of the log risk, and the area of the plotted point is inversely proportional to this variance. The TB vertical scale is compressed, due to the 20-fold mortality range. ‘Other’ includes all known medical causes except those categorised above. ‘Unspecified’ are non-specific causes.

**Extended Data Fig. 4:**
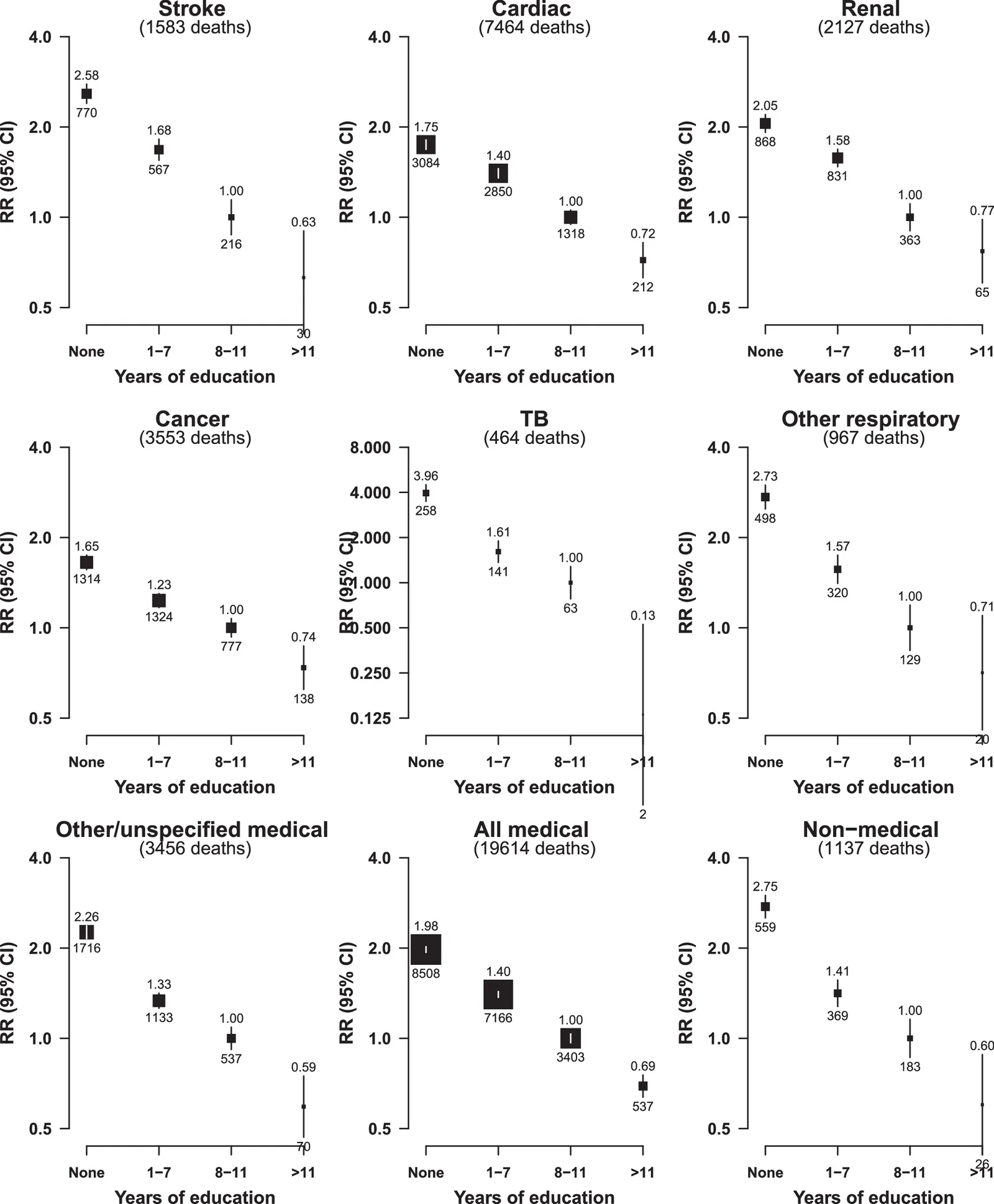
Associations of education with cause−specific mortality rate ratios at age 35 − 69 years among women who do not smoke or drink. Mortality rate ratios (RR) are standardised for age. Women with 8-11 years education had RR = 1; those with >11 years education had RR < 1 for each cause. The RR is shown above each square and the number of events below. For each group, the 95% CI reflects the variance of the log risk, and the area of the plotted point is inversely proportional to this variance. The TB vertical scale is compressed, due to the 20-fold mortality range. ‘Other’ includes all known medical causes except those categorised above. ‘Unspecified’ are non-specific causes.

For each of the nine underlying-cause-of-death categories (stroke, heart disease, renal disease, cancer, tuberculosis (TB), other respiratory disease, other medical causes, all medical causes and non-medical causes), there was a strong inverse association between educational level and mortality (Fig. 2), which was similar among men and women (Extended Data Figs. 3 and 4). Even after excluding the few with >11 years education (who had the lowest mortality), strong inverse associations remained. The mortality RRs comparing no schooling versus 8–11 years of education (the reference group in Fig. 2) were most extreme for respiratory TB (RR of 4.17, 95% CI 3.58–4.86) and other respiratory disease (RR of 3.35, 95% CI 2.87–3.92) but were about 2 for each of the other cause-of-death categories. These RRs, adjusted for age and sex, involved only participants who never smoked or drank.

**Fig. 3:**
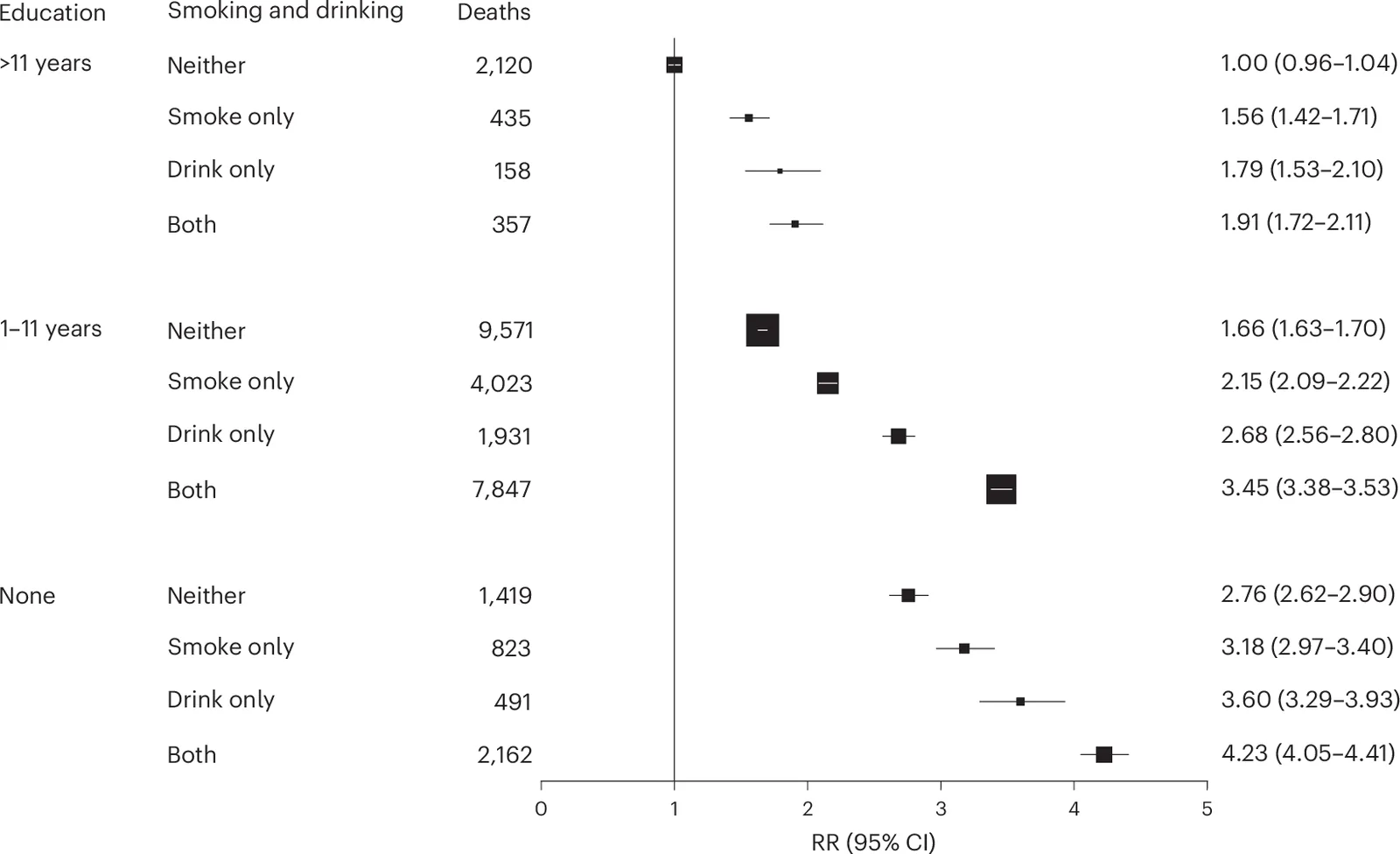
Joint associations of education, ever-smoking and ever-drinking with all-cause mortality RRs at ages 35–69 years, among men. Mortality RRs are standardized for age. Ever-smokers reported at baseline current or past use of cigarettes, bidis or (rarely) other forms of tobacco. Men with >11 years of education who neither smoked tobacco nor drank alcohol had an RR of 1. For each group, the 95% CI reflects the variance of the log risk in that group, and the area of the plotted point is inversely proportional to this variance.

Figure 3 shows all-cause mortality RRs for men with various combinations of smoking, drinking and education. At each educational level, the highest mortality was among men who both smoked and drank. The mortality RR comparing them with men who never smoked or drank was less extreme among men with no schooling than among those with tertiary education, but the absolute excess risks were similar, as is seen in Fig. 1b.

In the analyses of all-cause mortality adjusted for age, education and SES, men who both smoked and drank had almost twice the mortality rate of those who did neither (RR of 1.89, 95% CI 1.84–1.94); men who smoked had 1.26 (95% CI 1.22–1.29) times the mortality rate of those who never smoked in analyses adjusted for age, education, SES and drinking, and men who drank had 1.52 (95% CI 1.48–1.56) times the mortality rate of men who did not drink in analyses adjusted for age, education, SES and smoking (Supplementary Table 8). These RRs are less extreme than the RRs adjusted only for age but still accounted for 22% of all male mortality in the study population (Supplementary Table 9).

Most cigarette smokers used only cigarettes, most bidi smokers used only bidis and the mean daily consumption of cigarettes plus bidis at baseline was 10 (s.d. 5) among cigarette-only smokers, 14 (s.d. 8) among bidi-only smokers and 11 (s.d. 7) among all men who smoked (Table 1). The mean age men started smoking was 21.9 years (s.d. 4.2). The earlier education had ended, the earlier smoking started, the greater the mean daily consumption and the greater the use of bidis.

**Fig. 4:**
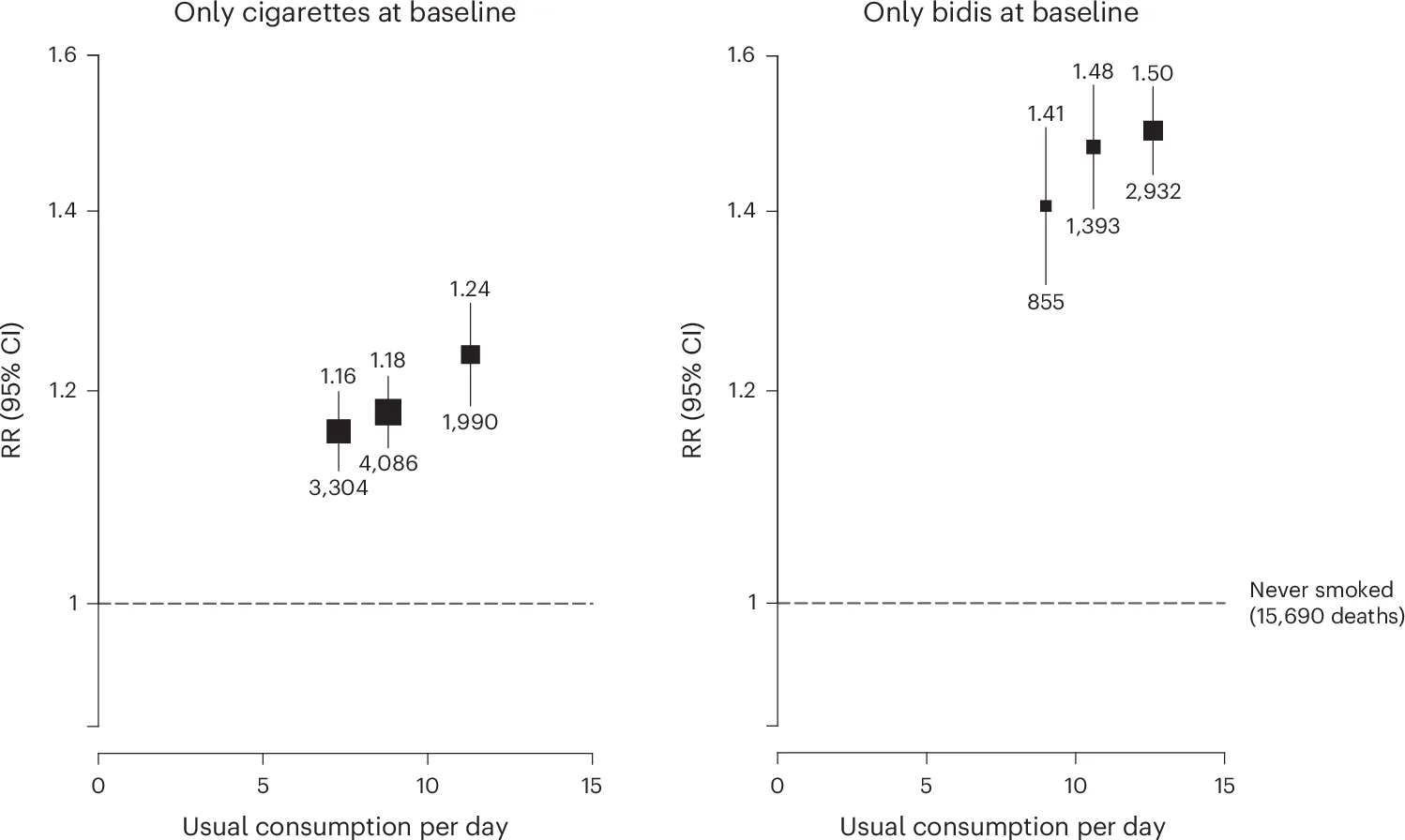
Associations of usual amount smoked with all-cause mortality RRs at ages 35–69 years, comparing men reporting current or past use of only cigarettes or only bidis at baseline versus men who never smoked. Mortality RRs are standardized for age, education and ever/never alcohol use and are from one fit of seven baseline-assigned groups: never-smokers, ever-smokers reporting at baseline current or past use of only cigarettes (<10, 10 or >10 per day) or only bidis (<10, 10 or >10 per day). The usual consumption (of the sum of cigarettes plus bidis) during follow-up in these baseline-assigned groups was estimated from a later resurvey (Supplementary Table 5). For each group, the 95% CI reflects the variance of the log risk of that group, and the area of the plotted point is inversely proportional to this variance. The 95% CI for never-smokers is 0.98–1.02.

**Extended Data Fig. 5:**
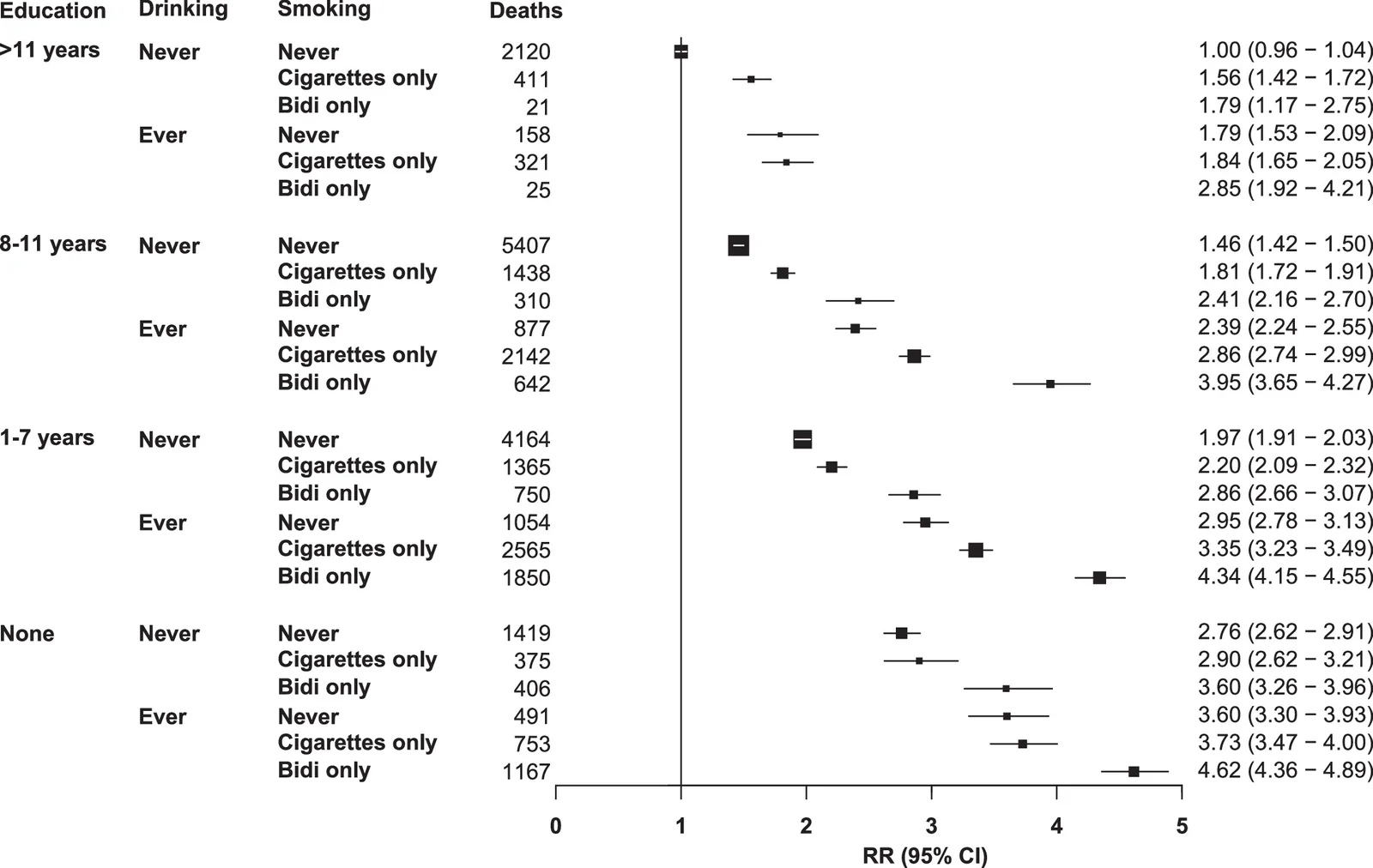
Joint associations of education, ever drinking and ever smoking of cigarettes only or bidis only with all-cause mortality rate ratios at ages 35-69 years, among men. Mortality rate ratios (RR) are standardised for age. Ever smokers were categorised into those who reported at baseline current or past use of only cigarettes of only bidis. Men with >11 years of education who did not drink alcohol and smoked neither cigarettes nor bidis had RR = 1. For each group, the 95% CI reflects the variance of the log risk in that group, and the area of the plotted point is inversely proportional to this variance.

**Extended Data Fig. 6:**
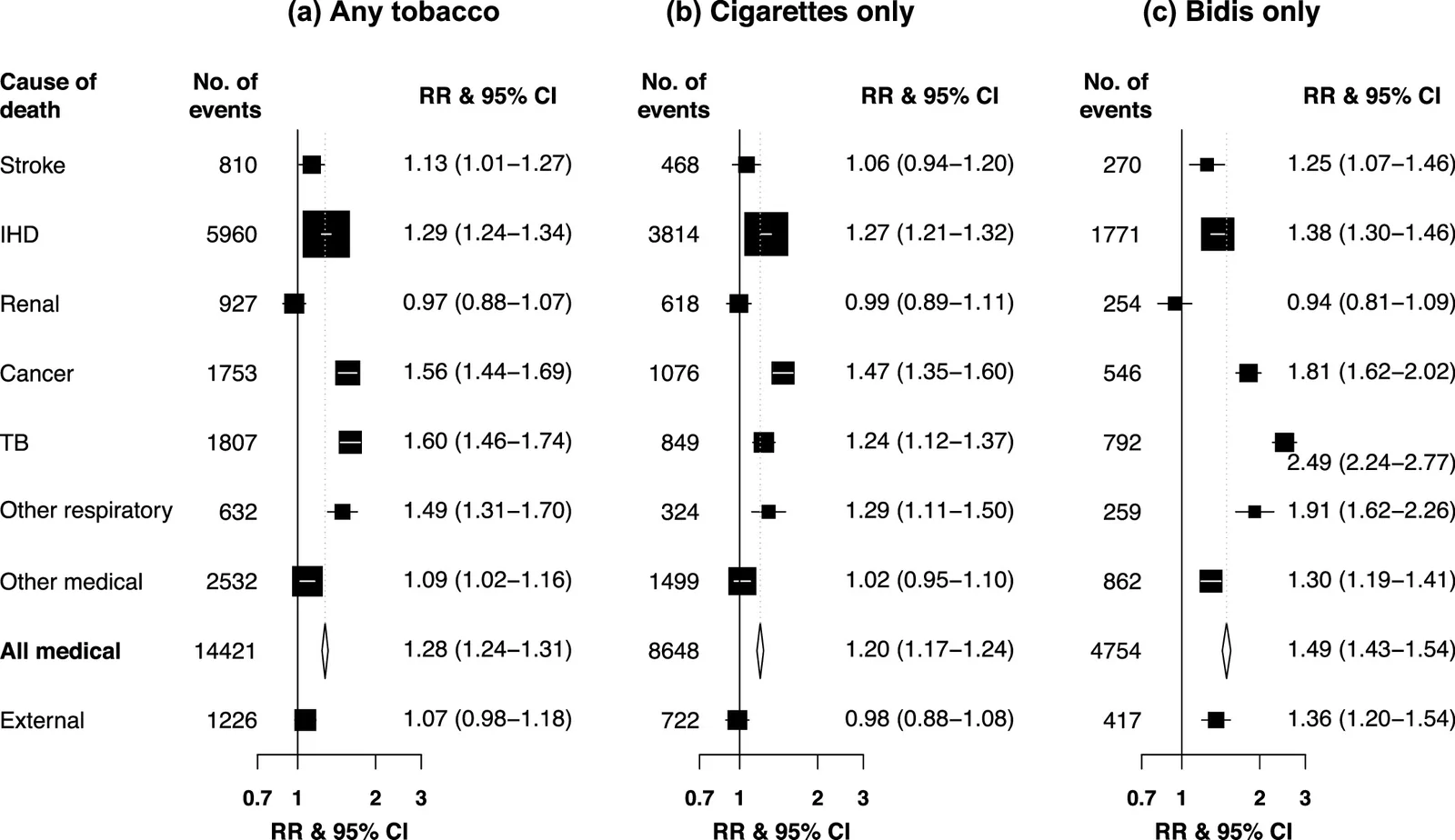
Associations of ever smoking of (a) Any tobacco; (b) Cigarettes only; or (c) Bidis only vs never-smokers with cause-specific mortality rate ratios at ages 35-69 years, among men. Mortality rate ratios (RR) are standardised for age, education and alcohol. For each group, the 95% CI reflects the variance of the log risk in that group, and the area of the plotted point is inversely proportional to this variance.

**Extended Data Fig. 7:**
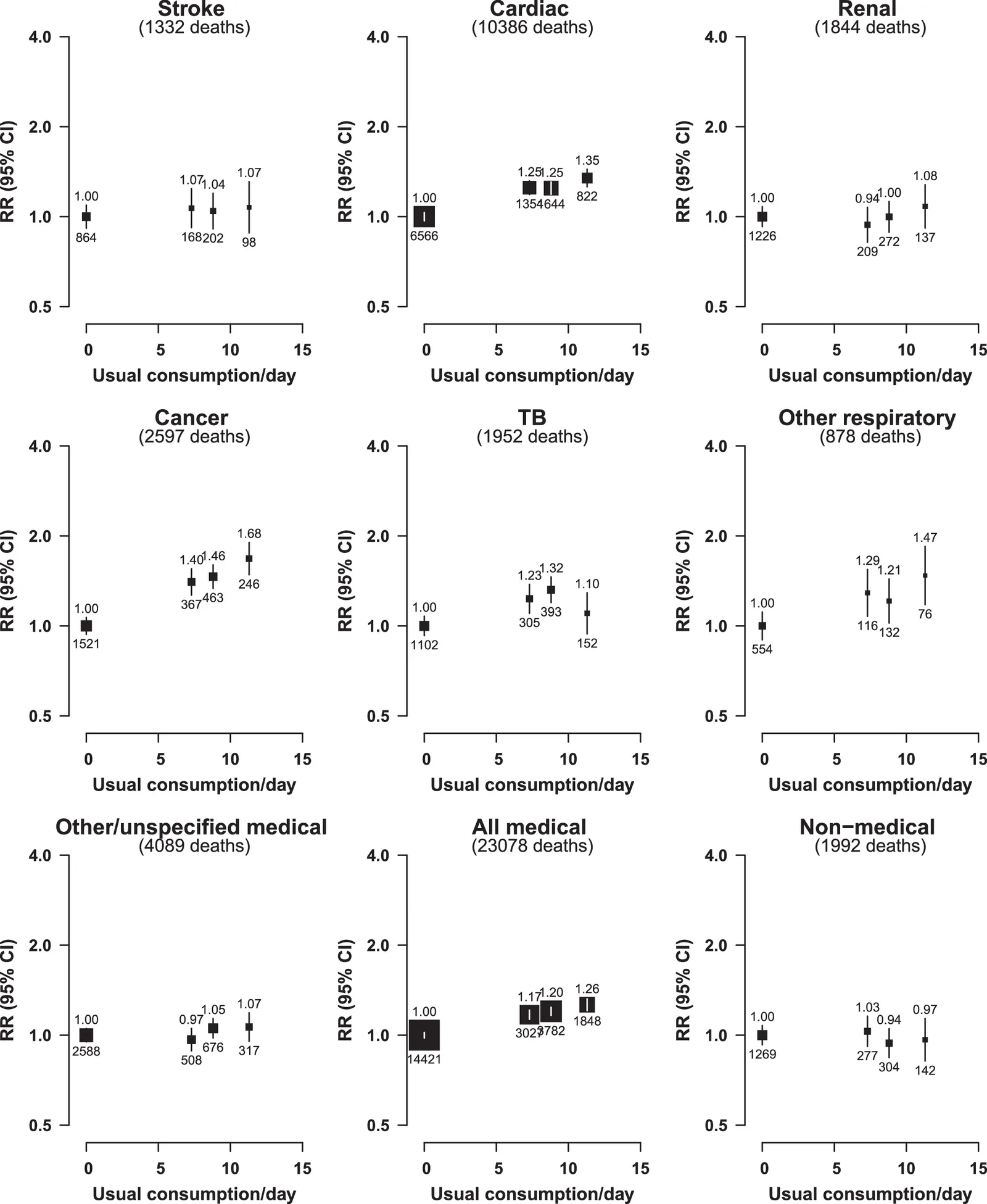
Associations of amount smoked with cause-specific mortality rate ratios at ages 35-69, comparing men reporting current or past use of only cigarettes at baseline vs never-smokers. Mortality rate ratios (RR) are standardised for age, education and ever/never alcohol drinking. Men who reported smoking other tobacco not shown. Men who never smoked cigarettes had RR = 1. For each group, the 95% CI reflects the variance of the log risk, and the area of the plotted point is inversely proportional to this variance. The RR is shown above each square and the number of events below. ‘Other’ includes all known medical causes except those categorised above. ‘Unspecified’ are non-specific causes.

**Extended Data Fig. 8:**
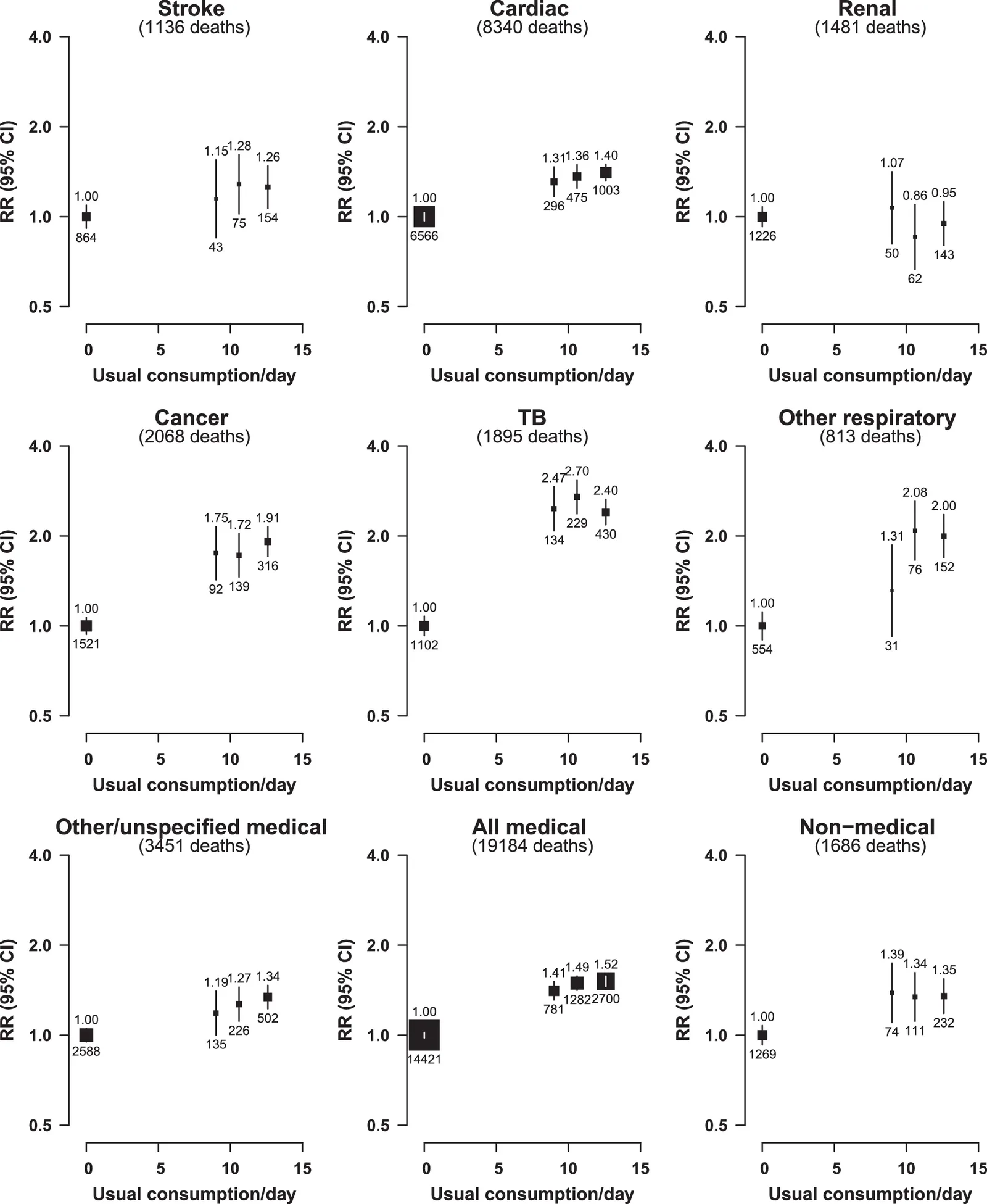
Associations of amount smoked with cause-specific mortality rate ratios at ages 35-69, comparing men reporting current or past use of only bidis at baseline vs never-smokers. Mortality rate ratios (RR) are standardised for age, education and ever/never alcohol drinking. Men who reported smoking other tobacco not shown. Men who never smoked bidis had RR = 1. For each group, the 95% CI reflects the variance of the log risk, and the area of the plotted point is inversely proportional to this variance. The RR is shown above each square and the number of events below. ‘Other’ includes all known medical causes except those categorised above. ‘Unspecified’ are non-specific causes.

Analyses adjusted for alcohol and education found positive associations of cigarette smoking, bidi smoking and any smoking with each of vascular, neoplastic and respiratory mortality, which accounted for the association of smoking with overall mortality (Extended Data Figs. 5 and 6). This smoking-associated excess mortality appeared to be approximately proportional to the usual daily consumption of cigarettes or of bidis (Fig. 4), despite the narrow range of usual consumption (Supplementary Table 7). Bidi smokers had higher RRs than cigarette smokers for all-cause mortality (Extended Data Fig. 5) and, particularly, respiratory mortality (Extended Data Figs. 6–8). Comparing bidi smokers directly with cigarette smokers, the all-cause mortality RR adjusted only for smoking frequency was 1.44 (95% CI 1.39–1.50; *χ*^2^_1_ = 368). If further adjusted for alcohol and education, however, this fell to 1.28 (95% CI 1.23–1.33; *χ*^2^_1_ = 149).

**Fig. 5:**
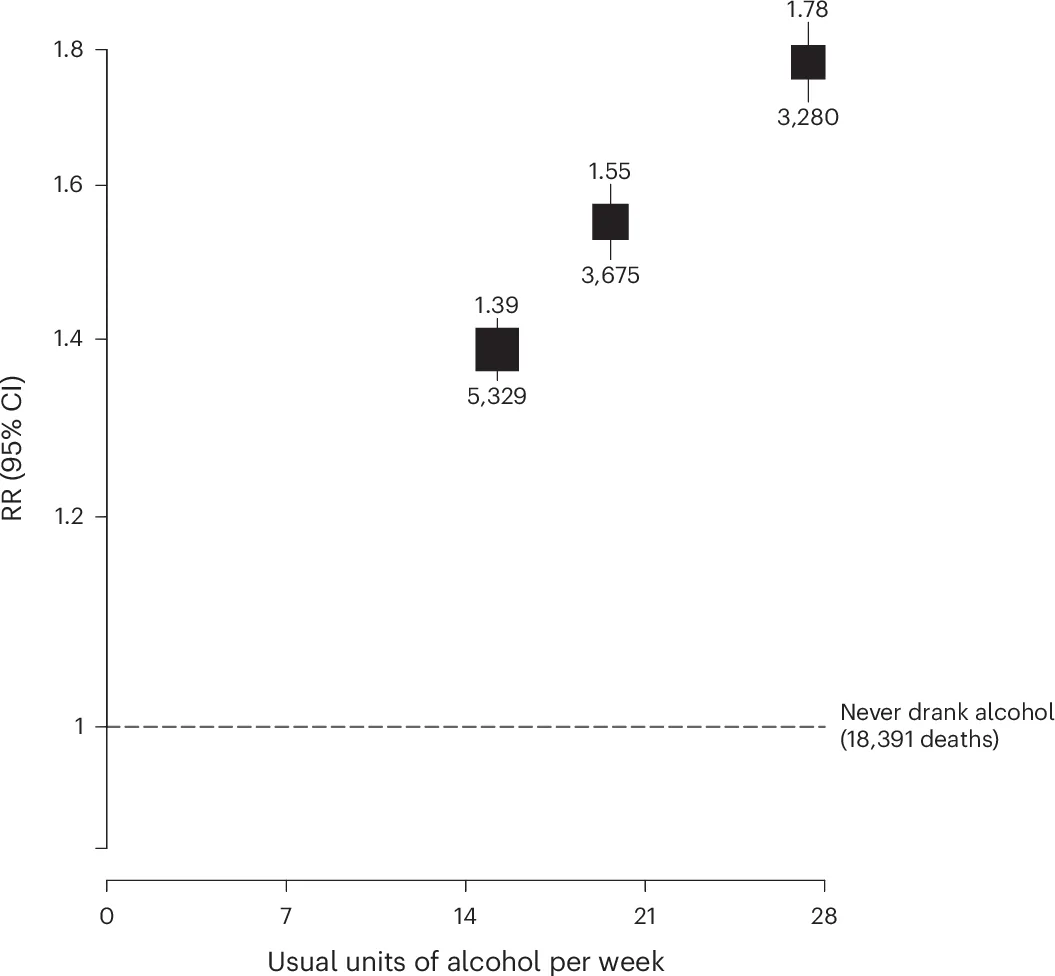
Associations of usual alcohol consumption (in 10-g units) with all-cause mortality RRs at ages 35–69 years, among men. Mortality RRs are standardized for age, education and ever/never smoked and are from four baseline-assigned groups: never drank alcohol or, at baseline, drank alcohol on <2, 2–6 or >6 days week^−1^. Usual consumption in these baseline-assigned groups was estimated from a later resurvey (Supplementary Table 6). For each group, the 95% CI reflects the variance of the log risk of that group, and the area of the plotted point is inversely proportional to this variance. The 95% CI for never-drinkers is 0.98–1.02.

**Extended Data Fig. 9:**
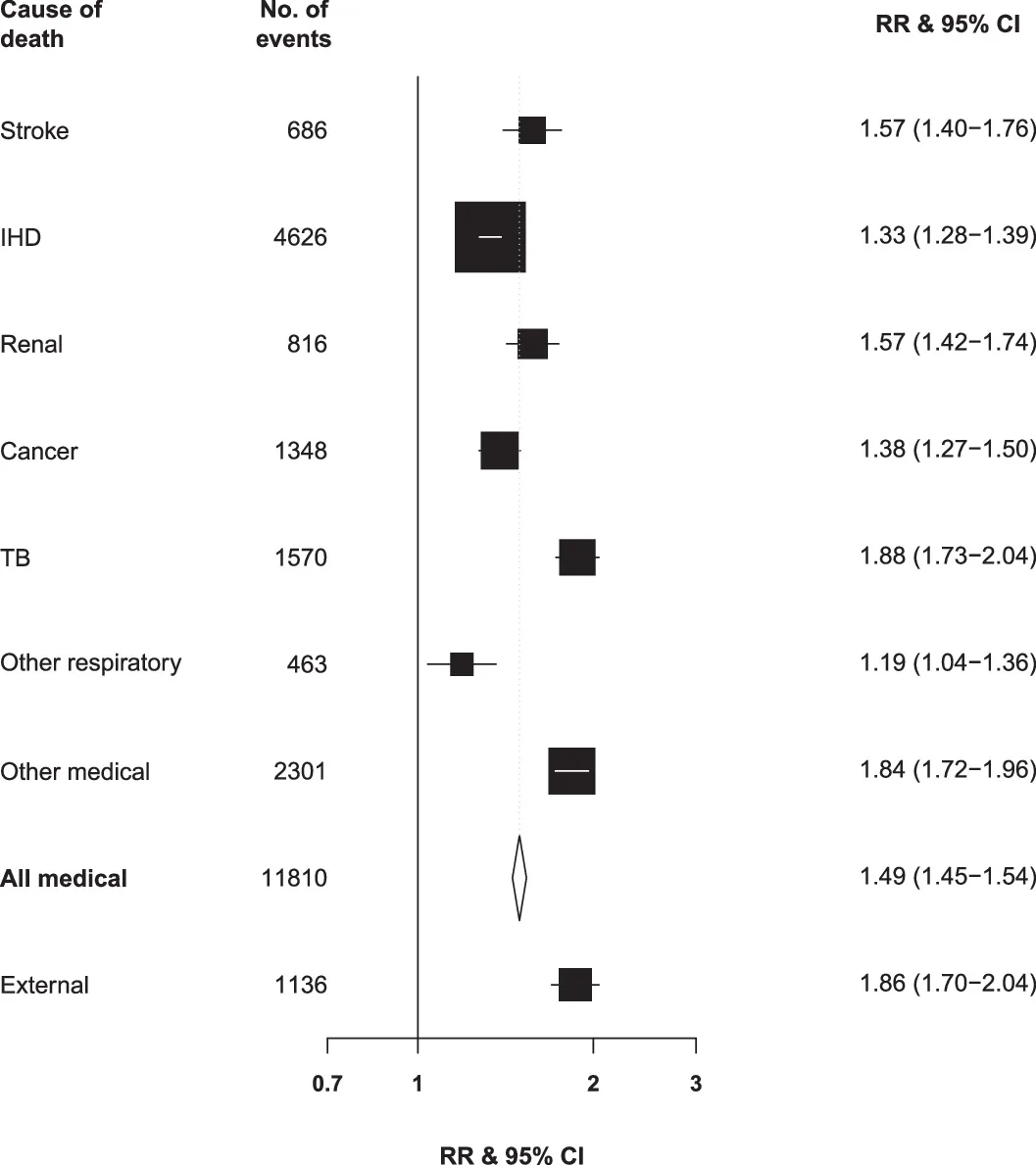
Associations of usual alcohol consumption (in 10gunits) with cause-specific mortality rate ratios at ages 35-69, among men. Mortality rate ratios (RR) are standardised for age, education and ever/never smoking. Ex-drinkers not shown. For each group, the 95% CI reflects the variance of the log risk in that group, and the area of the plotted point is inversely proportional o this variance. ‘Other’ includes all known medical causes except those categorised above. ‘Unspecified’ are non-specific causes.

**Extended Data Fig. 10:**
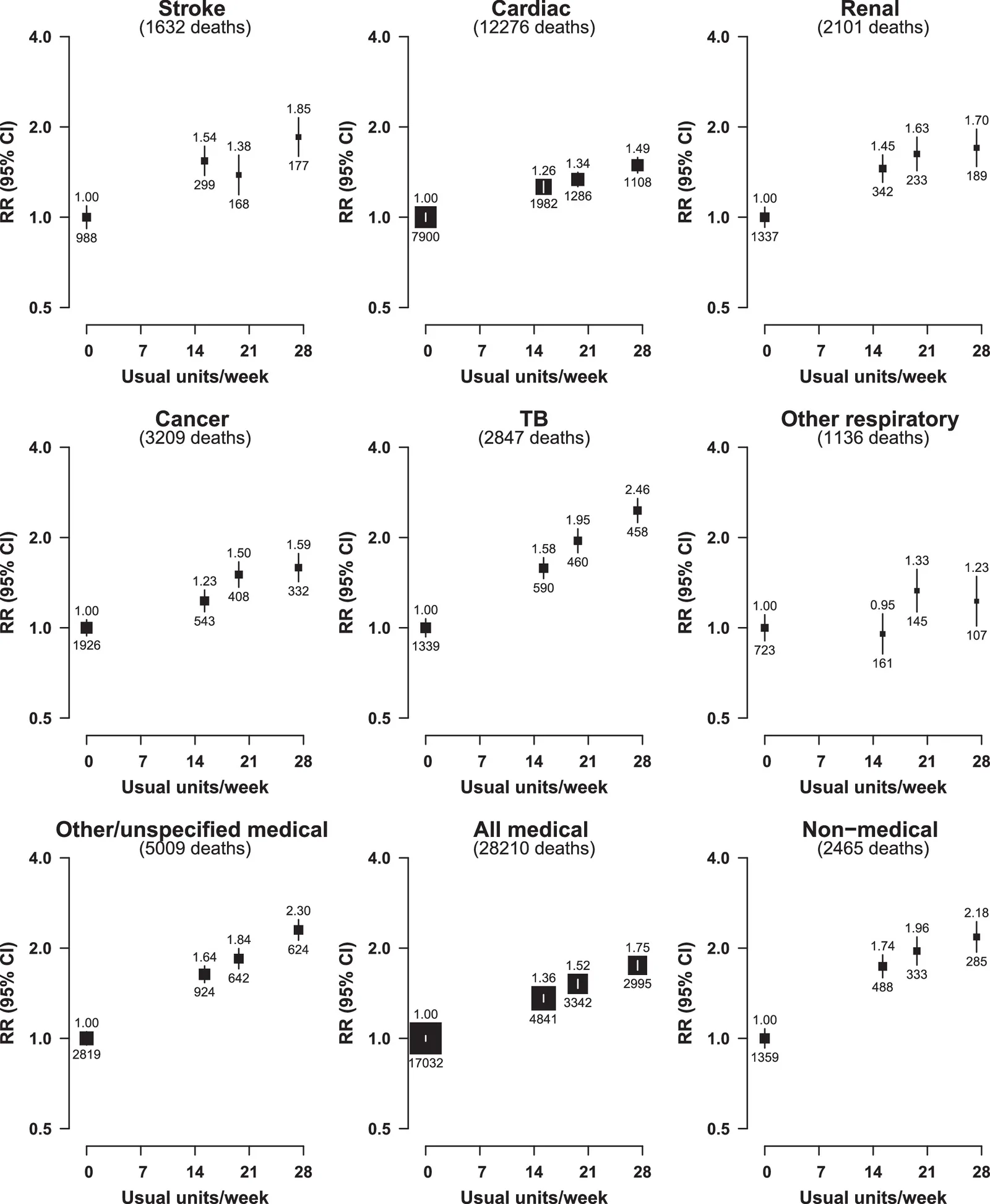
Associations of usual alcohol consumption (in 10g-units) with cause-specific mortality rate ratios at ages 35-69 years, among men. Mortality rate ratios (RR) are standardised for age, education and ever/never smoking. Ex-drinkers not shown. For each group, the 95% CI reflects the variance of the log risk in that group, and the area of the plotted point is inversely proportional o this variance. ‘Other’ includes all known medical causes except those categorised above. ‘Unspecified’ are non-specific causes.

At baseline, the mean alcohol consumption per drinker was 19 (s.d. 44) 10-g units per week. In analyses adjusted for smoking and education, overall mortality was positively associated with usual alcohol intake. A twofold difference in usual alcohol intake (14 units per week versus 28 units per week) corresponded to a twofold difference in the alcohol-associated excess mortality (39% versus 78% excess, based on RR of 1.39 versus RR of 1.78; Fig. 5). For all nine cause-of-death categories, mortality was higher among men who drank than men who did not drink, with no suggestion of any protective effect of moderate drinking against cardiac mortality (Extended Data Figs. 9 and 10).

## Discussion

In this urban Indian population, born around the mid-century, one in ten men and one in three women had had no schooling. Even among those who did not smoke or drink, those with no schooling had about three times the death rate in middle age (35–69 years) of those with tertiary education. Few women smoked or drank, but men who both smoked and drank had about double the death rate in middle age of those with neither habit. As men with no schooling were three times as likely to smoke or drink as men with tertiary education, smoking and drinking exacerbated the social inequalities in premature mortality. Although previous prospective studies in India have demonstrated associations of mortality with smoking, drinking and lower educational level or SES, they have not had sufficient power to quantify reliably the independent effects of these risk factors^13–19^.

The all-cause mortality RR of 1.26 associated with smoking among men is less extreme than in high-income settings^20–22^. However, mean consumption per smoker was only about 11 cigarettes or bidis per day, less than is now typical in high-income countries, and smoking began at mean age 22.4 years, which is somewhat later than is now typical^21,23^. This mortality RR is also less extreme than the mortality RR of 1.7 (with narrow confidence limits) reported in a large unmatched case–control study of a nationally representative sample of Indian deaths^15^. The present mortality RR of 1.26 (also with narrow limits) is, however, consistent with two smaller prospective studies in India, one in Mumbai^14^ and one south Asian but largely in India (PURE^16^). However, these comparisons are dependent upon the quantity smoked (which differed between studies) and duration (which was not consistently reported).

Risks associated with bidis (inexpensive, handmade; a small amount of tobacco wrapped in temburni leaf) are less well documented prospectively than risks from cigarettes. In this and other Indian studies^13,17^, bidis were associated with drinking and lack of education, and men smoking only bidis consumed higher numbers daily than those smoking only cigarettes. Therefore, although bidis were associated with higher risks than cigarettes (as in Mumbai^14^), this difference was so attenuated after standardizing for the daily number smoked, drinking and education as to suggest that residual confounding could largely or wholly account for whatever remained of it. Residual confounding cannot plausibly account for most of the difference between smokers and non-smokers but could well account for some of it.

For alcohol, the mortality RR comparing men who did and did not drink was 1.52 in analyses adjusted for smoking, SES and education, an excess of 52% (with narrow confidence limits) and a twofold difference in the usual amount drunk (mean 14 versus 28 10-g units per week) corresponded to a twofold difference in excess mortality (39% versus 78%). This RR of 1.52 among men who drank was greater than in three other largely or wholly Indian prospective studies (RR of 1.1 in Kerala, 1.2 in Mumbai and 1.3 in PURE^16,18,19^), although among heavier drinkers, the excess mortality was comparable in PURE and this study. The generally lower RRs in other Indian prospective studies of drinkers may reflect differences in usual alcohol intake, which are difficult to compare between studies or unexplained oddities in the small group who drank but did not smoke, as this small group strongly affects the alcohol RR.

Smoking and drinking are closely correlated; therefore, it is difficult to assess reliably their separate effects, especially because smoking or drinking patterns in men doing both may differ from those in men doing only one. Nevertheless, their joint effects are easier to assess reliably because the number doing both is large, as is the twofold mortality RR comparing them with men doing neither. If this excess mortality is mainly causal, about half the premature mortality in men who both smoke and drink is due to the combined effects of those habits. The attributable proportion may well, however, be somewhat less than half, as the confounding of smoking and drinking with lack of education is so strong that some residual confounding is likely to remain in education-adjusted analyses.

The absolute differences in mortality associated with smoking and drinking were similar in men with no schooling and with tertiary education; therefore, the proportional differences were greater in those with tertiary education. In Mumbai^18^ there were no strong interactions with years of education (≤8 versus >8 years) for drinking or cigarette smoking, but for bidi smoking, the RR was greater with more education. Most other studies were in high-income countries and did not report much interaction^6,7,9,11,12,18^.

Although our asset-based household SES quartiles were inversely associated with mortality, they were poorly reproducible at resurvey (partly reflecting the rapid pace of economic development in India) and of little independent relevance in analyses adjusted for educational level, smoking and drinking. By contrast, years of schooling were, for unclear reasons, remarkably strongly and independently predictive of mortality from each of the nine groups of causes, even among men and women who never smoked or drank. Inverse associations with many causes might well reflect major disparities in access to effective healthcare, but we cannot quantify these. The inverse association of educational level with mortality confirms^5^ that there are strong social determinants of health but does not identify their proximal causes or indicate that substantially more education would substantially reduce the disparities. It was stronger in this study than in high-income settings^24,25^, perhaps reflecting greater social disparities in Chennai^26^ or greater disparities in healthcare^27^.

It may, however, not be necessary to understand the exact mechanisms of social inequalities in health to reduce them, partly by widespread access to effective healthcare and partly by addressing the wider determinants of health (for example, education, housing and lifestyle factors). As India developed over the past half-century, the probability that a 50-year-old would die before age 70 years decreased from about half at 1970 death rates (relating to people born in the first quarter of the century) to only about one third at 2010 rates^28^ (relating to those born around the mid-century, as in our study); therefore, further substantial progress can reasonably be anticipated, especially if access to effective healthcare improves^29^.

The chief strengths of this study are the large sample size, quality control of the baseline survey, availability of a partial resurvey (allowing assessment of the long-term relevance of the information collected at baseline), active follow-up of deaths (ensuring near-completeness, except in demolished areas), verbal autopsy methods (allowing better assessment of causes of death than from official records and reducing the number of unspecific and unknown causes of death), the experience of the Chennai-based researchers in conducting large-scale epidemiological studies (which also accounts for the study’s location) and exclusion of individuals with chronic disease at recruitment (helping avoid biases owing to reverse causality). Although the main mortality analyses used ever-smoking and ever-drinking only at baseline, the sample resurvey showed these rarely varied, limiting any adverse consequences of our inability to account for time-varying exposures and confounding in the absence of repeat resurveys of the full study population. Most men who smoked also drank, most men who drank also smoked and the number doing both was substantially larger than the numbers doing only one. The risk associated with doing both can therefore be assessed reliably. By contrast, the subdivision of this risk into two parts, one associated with smoking and one with drinking, is less reliable, as it depends on the risks in the smaller groups with only one habit (and on assumptions about the independence of their effects). As smoking, drinking and lack of education are correlated, some of the adjusted risk associated with smoking or drinking may well reflect residual or unmeasured confounding.

Major limitations are that no blood or other biological material was stored; therefore, no analyses of genetic or biochemical factors are possible, and little information was collected on physical activity or dietary factors. Verbal autopsy methods did not distinguish between several causes of death (such as ischaemic and haemorrhagic stroke), and analyses describe only mortality not incidence. Moreover, apart from smoking and drinking, the correlates of education that are directly or indirectly responsible for its inverse association with mortality are largely unknown; in particular, no information was collected on caste, income or access to healthcare.

In this population, very low body mass index (BMI <20 kg m^−2^) was associated with lack of education and increased all-cause mortality^30^. Blood pressure was not strongly associated with educational level, although it was strongly related to mortality; each 20-mm Hg difference in usual systolic blood pressure (SBP) was associated with a twofold difference in vascular mortality^30^, as in Western populations^31^.

Perhaps less importantly, the population is not representative of Chennai (as it omits homeless or transient people and may somewhat underrepresent the wealthy), of all Indian cities (as smoking and drinking prevalences vary, although Chennai is not exceptional in the context of India’s major cities with respect to educational opportunities or the prevalence of major risk factors) or of rural India. The qualitative findings should, however, be relevant throughout India and in many other middle-income settings^2,4^: social inequalities in adult mortality are remarkably steep, for unclear reasons, and smoking, drinking and, particularly, their combination exacerbate these differences.

Realistic strategies for limiting mortality from smoking and drinking should recognize that, at least for this generation in this population, virtually none of the men who were smoking or drinking in about the year 2000 actually stopped over the next decade or more, despite the strong global and national pressures to change behaviour. Increased taxation could help^32^ but could also exacerbate inequalities. How, realistically, can men best be protected from continuing and women from starting? Even more importantly, how can realistic strategies be found for sustainably improving the delivery of effective healthcare, particularly to those most in need of it? At present, the main reasons for the extraordinarily strong relationships in this study between educational status and adult mortality remain unclear and need to be better understood.

## Methods

### Ethics statement

This is a historical study involving questionnaires, physical measurements and active follow-up (with no biological material collected from the study participants). The study research proposal was approved in 1997 by the Cancer Institute (WIA) in Chennai, ensuring that the study fulfilled the criteria required by the Indian Council of Medical Research regarding the treatment of human subjects in medical research. The Chennai Epidemiological Research Center’s Institutional Ethics Committee (IEC) now has ethical oversight for the study. Secondary analyses of anonymized data have been approved by the IEC and Oxford’s Tropical Research Ethics Committee.

#### Study design, participants, and procedures

The study design, population and methods have been described previously^30,33^. The study took place in the south Indian city of Chennai (Supplementary Fig. 1). In the baseline survey, from 1 January 1998 to 31 December 2001, fieldworkers sought to visit all households in two of the city’s ten administrative zones and all in either 30% or 50% of the streets (randomly selected) in five of the remaining eight zones. All household members aged ≥35 years were invited to participate; 5% (25,184 out of 525,994) of those present declined, and 500,810 were recruited after providing informed consent (which was verbal, to be fully inclusive of those with minimal schooling) to the in-person interview and to passive (through public municipal records) and active follow-up. Trained fieldworkers administered a questionnaire (Supplementary Information pages 2–4) that included occupation (but not income), household indicators of SES and, for each participant in the household, demographic characteristics, educational level, tobacco smoking habits, alcohol drinking habits, selected dietary factors, medical history and the participant’s children’s names (for verification during any follow-up of fieldwork reliability and participant identity). Physical measurements included height, weight, peak expiratory flow rate and blood pressure but no invasive tests. Weekly throughout the baseline survey, 5% of recent interviewees were randomly selected for prompt re-interview and remeasurement by a fieldwork supervisor for quality control and fieldworker training and discipline. To help assess reproducibility and usual intake levels, a resurvey was undertaken in 2013–2014, about 14 years after the baseline survey, of 10,161 participants in randomly selected streets in the study areas, using the same procedures and precautions.

Educational level was self-reported as years of formal education, later categorized as none, 1–7, 8–11 or >11 (tertiary). A composite household-asset-based SES score was obtained by summing scores on the basis of several self-reported indicators—housing type and number of rooms; housing ownership; type of cooking fuel; and household ownership of a bicycle, motorcycle, car, refrigerator, television or electric grain grinder (but not individual characteristics, for example, caste, religion, occupation or income)—and the score was grouped into quartiles. Data were self-reported on smoking (each of cigarettes, bidis or other) and chewing (various products) and included status (never, ex or current), frequency (times per day) and ages at starting and stopping. Data were self-reported on drinking each of various alcoholic products including status, frequency (days per week), quantity on days when drinking and ages at starting and stopping, from which weekly intake was estimated in standard 10-g units of alcohol. For smoking and for drinking, the main analyses compared ever- versus never-users, as cessation (before or after baseline) was uncommon (Results).

Mortality was monitored by active and passive follow-up to 2020, when coronavirus disease 2019 intervened. Active follow-up involved visits to all houses (annually to 2005 and then biennially) by trained non-medical graduate fieldworkers. Participant identity and fieldwork integrity was verified by fieldworkers recording again participants’ children’s names, blinded to the baseline information. If participants had moved, information was sought from neighbours about when and where to. For those moving elsewhere in Chennai, follow-up was attempted at the new address. Those who could not be contacted or traced (generally because of area demolition for redevelopment) were monitored by passive follow-up. This used manual and computerized probabilistic linkage to city mortality records, considering name, age, sex, address and name of father or spouse, seeking confirmation of probable matches by fieldworker visits.

Causes of death were sought by verbal autopsy, using fieldworker interviews with families or other informants^34,35^. An open narrative, supplemented by a short checklist, yielded a one-page chronological description, written in the field, of any signs or symptoms and any treatment received before death. Again for training, quality control and discipline, a randomly selected 5% sample of narratives were repeated by a supervisor visiting informants a week after the fieldworker^35^. Each written narrative was assessed by two study doctors independently assigning a three-character ICD-10 code for the probable underlying cause (Supplementary Table 1), then discussing and resolving any discrepancies.

#### Statistical analyses

The main analyses excluded participants with chronic disease (myocardial infarction, stroke, cancer, asthma or TB) to avoid or limit reverse causality and are of mortality at ages 35–69 years, because verbal autopsy arrives at the probable underlying cause of death reliably in middle age (7% of deaths before 70 years were from unspecified causes in this study; Supplementary Table 1) but less so at older ages (42% of deaths at 70 years or older were from unspecified causes)^34,35^. Follow-up (from recruitment) was censored at 1 January 2020, emigration from Chennai or death.

Cox regression analyses were used to estimate RRs by educational level (four categories), SES score (quartiles), smoking (ever or never) and drinking (ever or never), stratified by 5-year groups of age at risk from 35–69 years (censoring at age 70 years), sex and, where appropriate, adjusted for each other. Although the ratio of the mortality rates in two categories may vary during follow-up, Cox regression can still be used to estimate an overall mortality RR, that is, effectively averaged over the whole follow-up period (without requiring an exactly constant ‘proportional hazard’). Ages were recorded in completed years (thus, 0.5 was added to mean ages).

Where there are four categories, Cox regression arbitrarily designates one the reference group and yields the variances and co-variances of the log RR values for the three others. From them, the variance of the log mortality rate in each of the four groups (one being the reference group) was estimated^36^. These do not depend on which group was arbitrarily designated the reference group, and each approximates the inverse of the number of deaths in its group. If the variance of the log mortality rate in one group is *V*, the 95% CI plotted for that group’s RR runs from RR × exp(−1.96√*V*) to RR × exp(1.96√*V*). For the reference group (with RR of 1) as for other groups, its CI then describes how chance could have affected the log mortality rate in that one group.

Participants resurveyed after about 14 years were categorized by smoking habits reported at baseline (past or current <10, 10 or >10 cigarettes or bidis per day), and for each baseline-assigned category mean consumption per day at resurvey yielded the estimated usual cigarette or bidi consumption during follow-up among all study participants in that category (not just those resurveyed). Similarly for alcohol, resurveyed participants were categorized by drinking habits reported at baseline (past or current ≤1, 2–6 or 7 days week^−1^), and for each baseline-assigned category, mean intake per week at resurvey yielded the estimated usual alcohol intake during follow-up in all participants. All-cause mortality RRs (estimated from all participants and adjusted for age, education and alcohol or smoking) in these baseline-assigned smoking or drinking categories were plotted against usual consumption, thereby allowing fully for the ‘regression dilution bias’ that can result from random errors or within-person temporal variability in baseline measurements when assessing associations between an exposure and disease^37^.

The relevance of education, smoking and drinking to the probability of a 35-year-old dying before age 70 years was assessed by combining the risk factor prevalences and age-adjusted RRs in this study with age- and sex-specific all-cause mortality rates from Chennai official statistics^38^. The probability of a 35-year-old dying before age 70 years was calculated by multiplying the RRs by a common constant to make their inverse-variance-weighted average match the uniformly age-standardized death rate per 1,000 person-years at ages 35–69 years (that is, the simple mean of the seven age-specific rates at ages 35–39 years to 65–69 years) in urban Tamil Nadu in 2010 (the median year of death in the study)^39^.

The analyses used SAS (v9.4) and R (v4.1.2).

#### Reporting summary

Further information on research design is available in the Nature Portfolio Reporting Summary linked to this article.

## Supplementary information

Supplementary Information Supplementary Fig. 1, Supplementary Tables 1–9, questionnaire and extended abstract.

Reporting summary

Peer Review File

## Data Availability

The primary data from this study are not publicly available because this was not part of the informed consent and therefore did not enable public data sharing. However, deidentified data may be made available upon reasonable request to S.L. (sarah.lewington@ndph.ox.ac.uk) according to the principles set out in the Oxford Population Health’s data sharing policy and following approval by the relevant institutional and ethics committees. Requests will be considered, reviewed and actioned within 6 months of receipt.

## Code availability

The analytical coding is available via GitHub at https://github.com/PaulSherliker/Global-Population-Studies/tree/Cox-Macro and https://github.com/PaulSherliker/Global-Population-Studies/tree/Chennai-Study. Further code can be made available on reasonable request to S.L. (sarah.lewington@ndph.ox.ac.uk). Requests will be considered, reviewed and actioned within 6 months of receipt.

## References

[1] Petrovic, D. et al. The contribution of health behaviors to socioeconomic inequalities in health: a systematic review. *Prev. Med.* **113**, 15–31 (2018). 10.1016/j.ypmed.2018.05.003 29752959

[2] Allen, L. et al. Socioeconomic status and non-communicable disease behavioural risk factors in low-income and lower-middle-income countries: a systematic review. *Lancet Glob. Health* **5**, 277–289 (2017). 10.1016/S2214-109X(17)30058-X PMC5673683 28193397

[3] Garrett, B. E., Martell, B. N., Caraballo, R. S. & King, B. A. Socioeconomic differences in cigarette smoking among sociodemographic groups. *Prev. Chronic Dis.* **16**, 180553 (2019). 10.5888/pcd16.180553 PMC6583815 31198164

[4] Collins, S. E. Associations between socioeconomic factors and alcohol outcomes. *Alcohol Res.* **38**, 83–94 (2016). 10.35946/arcr.v38.1.11 PMC4872618 27159815

[5] Marmot, M. & Wilkinson, R. *Social Determinants of Health* 2nd edn (Oxford Univ. Press, 2005).

[6] de Mestral, C., Bell, S., Stamatakis, E. & Batty, G. D. Testing differential associations between smoking and chronic disease across socioeconomic groups: pooled data from 15 prospective studies. *Epidemiology* **30**, 48–51 (2019). 10.1097/EDE.0000000000000922 PMC6278911 30247206

[7] Charafeddine, R., Van Oyen, H. & Demarest, S. Does the association between smoking and mortality differ by educational level? *Soc. Sci. Med.* **74**, 1402–1406 (2012). 10.1016/j.socscimed.2012.01.015 22401648

[8] Gruer, L., Hart, C. L., Gordon, D. S. & Watt, G. C. Effect of tobacco smoking on survival of men and women by social position: a 28 year cohort study. *Br. Med. J.* **338**, b480 (2009). 10.1136/bmj.b480 PMC2645845 19224884

[9] Lewer, D., McKee, M., Gasparrini, A., Reeves, A. & de Oliveira, C. Socioeconomic position and mortality risk of smoking: evidence from the English Longitudinal Study of Ageing (ELSA). *Eur. J. Public Health* **27**, 1068–1073 (2017). 10.1093/eurpub/ckx059 PMC5881724 28481981

[10] Nielsen, N. R., Schnohr, P., Jensen, G. & Grønbaek, M. Is the relationship between type of alcohol and mortality influenced by socio-economic status? *J. Intern. Med.* **255**, 280–288 (2004). 10.1046/j.1365-2796.2003.01268.x 14746566

[11] Schnohr, C. et al. Does educational level influence the effects of smoking, alcohol, physical activity, and obesity on mortality? A prospective population study. *Scand. J. Public Health* **32**, 250–256 (2004). 10.1080/14034940310019489 15370764

[12] Marang-van de Mheen, P. J., Smith, G. D. & Hart, C. L. The health impact of smoking in manual and non-manual social class men and women: a test of the Blaxter hypothesis. *Soc. Sci. Med.* **48**, 1851–1856 (1999). 10.1016/s0277-9536(99)00071-4 10405021

[13] Duong, M. et al. Effects of bidi smoking on all-cause mortality and cardiorespiratory outcomes in men from south Asia: an observational community-based substudy of the Prospective Urban Rural Epidemiology Study (PURE). *Lancet Glob. Health* **5**, e168–e176 (2017). 10.1016/S2214-109X(17)30004-9 28104186

[14] Gupta, P. C., Pednekar, M. S., Parkin, D. M. & Sankaranarayanan, R. Tobacco associated mortality in Mumbai (Bombay) India. Results of the Bombay Cohort Study. *Int. J. Epidemiol.* **34**, 1395–1402 (2005). 10.1093/ije/dyi196 16249218

[15] Jha, P. et al. A nationally representative case–control study of smoking and death in India. *N. Engl. J. Med.* **358**, 1137–1147 (2008). 10.1056/NEJMsa0707719 18272886

[16] Joseph, P. et al. Cardiovascular disease, mortality, and their associations with modifiable risk factors in a multi-national South Asia cohort: a PURE substudy. *Eur. Heart J.* **43**, 2831–2840 (2022). 10.1093/eurheartj/ehac249 35731159

[17] Mishra, S. et al. Trends in bidi and cigarette smoking in India from 1998 to 2015, by age, gender and education. *BMJ Glob. Health* **1**, e000005 (2016). 10.1136/bmjgh-2015-000005 PMC5321300 28588906

[18] Pednekar, M. S., Vasa, J., Narake, S. S., Sinha, D. N. & Gupta, P. C. Tobacco and alcohol associated mortality among men by socio-economic status in India. *Epidemiol. Open J.* **1**, 2–15 (2016).

[19] Ramadas, K. et al. Effect of tobacco chewing, tobacco smoking and alcohol on all-cause and cancer mortality: a cohort study from Trivandrum, India. *Cancer Epidemiol.* **34**, 405–412 (2010). 10.1016/j.canep.2010.04.006 20444665

[20] Doll, R., Peto, R., Boreham, J. & Sutherland, I. Mortality in relation to smoking: 50 years’ observations on male British doctors. *Br. Med. J.* **328**, 1519 (2004). 10.1136/bmj.38142.554479.AE PMC437139 15213107

[21] Pirie, K., Peto, R., Reeves, G. K., Green, J. & Beral, V. The 21st century hazards of smoking and benefits of stopping: a prospective study of one million women in the UK. *Lancet* **381**, 133–141 (2013). 10.1016/S0140-6736(12)61720-6 PMC3547248 23107252

[22] Thun, M., Peto, R., Boreham, J. & Lopez, A. D. Stages of the cigarette epidemic on entering its second century. *Tob. Control* **21**, 96–101 (2012). 10.1136/tobaccocontrol-2011-050294 22345230

[23] Banks, E. et al. Tobacco smoking and all-cause mortality in a large Australian cohort study: findings from a mature epidemic with current low smoking prevalence. *BMC Med.* **13**, 38 (2015). 10.1186/s12916-015-0281-z PMC4339244 25857449

[24] Huisman, M. et al. Educational inequalities in cause-specific mortality in middle-aged and older men and women in eight western European populations. *Lancet* **365**, 493–500 (2005). 10.1016/S0140-6736(05)17867-2 15705459

[25] Steenland, K., Henley, J. & Thun, M. All-cause and cause-specific death rates by educational status for two million people in two American Cancer Society cohorts, 1959–1996. *Am. J. Epidemiol.* **156**, 11–21 (2002). 10.1093/aje/kwf001 12076884

[26] Gini index (World Bank estimate). *The World Bank* https://data.worldbank.org/indicator/SI.POV.GINI?name_desc=false (accessed 2 November 2020).

[27] Balarajan, Y., Selvaraj, S. & Subramanian, S. V. Health care and equity in India. *Lancet* **377**, 505–515 (2011). 10.1016/S0140-6736(10)61894-6 PMC3093249 21227492

[28] Norheim, O. F. et al. Avoiding 40% of the premature deaths in each country, 2010-30: review of national mortality trends to help quantify the UN sustainable development goal for health. *Lancet* **385**, 239–252 (2015). 10.1016/S0140-6736(14)61591-9 25242039

[29] Sen, G., Iyer, A. & George, A. Structural reforms and health equity: a comparison of NSS surveys, 1986–87 and 1995–96. *Econ. Polit. Wkly* **37**, 1342–1385 (2002).

[30] Gajalakshmi, V. et al. Body-mass index, blood pressure, and cause-specific mortality in India: a prospective cohort study of 500,810 adults. *Lancet Glob. Health* **6**, 787–794 (2018). 10.1016/S2214-109X(18)30267-5 PMC6005121 29903379

[31] Lewington, S., Clarke, R., Qizilbash, N., Peto, R. & Collins, R. Age-specific relevance of usual blood pressure to vascular mortality: a meta-analysis of individual data for one million adults in 61 prospective studies. *Lancet* **360**, 1903–1913 (2002). 10.1016/s0140-6736(02)11911-8 12493255

[32] Jha, P. & Peto, R. Global effects of smoking, of quitting, and of taxing tobacco. *N. Engl. J. Med.* **370**, 60–68 (2014). 10.1056/NEJMra1308383 24382066

[33] Gajalakshmi, V., Peto, R., Kanimozhi, V. C., Whitlock, G. & Veeramani, D. Cohort profile: the Chennai prospective study of mortality among 500,000 adults in Tamil Nadu, South India. *Int. J. Epidemiol.* **36**, 1190–1195 (2007). 10.1093/ije/dym091 17510070

[34] Gajalakshmi, V. & Peto, R. Verbal autopsy of 80,000 adult deaths in Tamil Nadu, South India. *BMC Public Health* **4**, 47 (2004). 10.1186/1471-2458-4-47 PMC535898 15488138

[35] Gajalakshmi, V., Peto, R., Kanaka, S. & Balasubramanian, S. Verbal autopsy of 48,000 adult deaths attributable to medical causes in Chennai (formerly Madras), India. *BMC Public Health* **2**, 7 (2002). 10.1186/1471-2458-2-7 PMC113750 12014994

[36] Easton, D. F., Peto, J. & Babiker, A. G. Floating absolute risk: an alternative to relative risk in survival and case–control analysis avoiding an arbitrary reference group. *Stat. Med.* **10**, 1025–1035 (1991). 10.1002/sim.4780100703 1652152

[37] Clarke, R. et al. Underestimation of risk associations due to regression dilution in long-term follow-up of prospective studies. *Am. J. Epidemiol.* **150**, 341–353 (1999). 10.1093/oxfordjournals.aje.a010013 10453810

[38] *Death Rates for Urban Tamil Nadu* (Registrar General of India, 2015).

[39] Peto, R., Watt, J. & Boreham, J. Deaths from smoking. *UICC* https://www.deathsfromsmoking.net/ (accessed 6 February 2026).

