# Supplementary material for: Combined impact of smoking, alcohol and social inequalities on premature mortality in urban India
Source: Nat Health. 2026 Jun 19;1(10):1082–91. doi: 10.1038/s44360-026-00148-4 (PMC13630575; doi:10.1038/s44360-026-00148-4)
Supplement: Supplementary file 1 [file 44360_2026_148_MOESM1_ESM.pdf]

nature health

Supplementary information

https://doi.org/10.1038/s44360-026-00148-4

Combined impact of smoking, alcohol and social inequalities on premature mortality in urban India

In the format provided by the authors and unedited

Supplementary Information

Table of contents

		Page
**Supplementary Figure 1**	Map showing locations of (a) the city of Chennai within Tamil Nadu, and (b) study areas within Chennai	1
**Questionnaire**	English-language translation of Tamil-language baseline survey questionnaire	2-4
**Supplementary Table 1**	Categorisation of ICD-10 codes for death causes, and category-specific numbers of deaths	5
**Supplementary Table 2**	Numbers of participants recruited and numbers excluded, by reason for exclusion and by sex	6
**Supplementary Table 3**	Age dependence of baseline characteristics, by sex	7-8
**Supplementary Table 4**	Education and SES at baseline and at resurvey about 14 years later, by sex	9
**Supplementary Table 5**	Smoking and drinking at baseline and at resurvey about 14 years later, by sex	10
**Supplementary Table 6**	Smoking at baseline versus that at resurvey among men aged 35-59 at entry and resurveyed 14 years later	11
**Supplementary Table 7**	Drinking at baseline versus that at resurvey among men aged 35-59 at entry and resurveyed 14 years later	12
**Supplementary Table 8**	Men: Effects of sequential adjustment for confounders on the all-cause mortality rate ratios for smoking and/or drinking	13
**Supplementary Table 9**	Numbers of deaths among men associated with only smoking, only drinking, or both	14
**Extended abstract**		15

Supplementary Figure 1: Map showing locations of (a) the city of Chennai within Tamil Nadu, and (b) study areas within Chennai

(a) States of South India
(Tamil Nadu 2001 population: 62 million)

(b) Chennai City administrative zones
(Chennai City 2001 population*: 4.2 million)

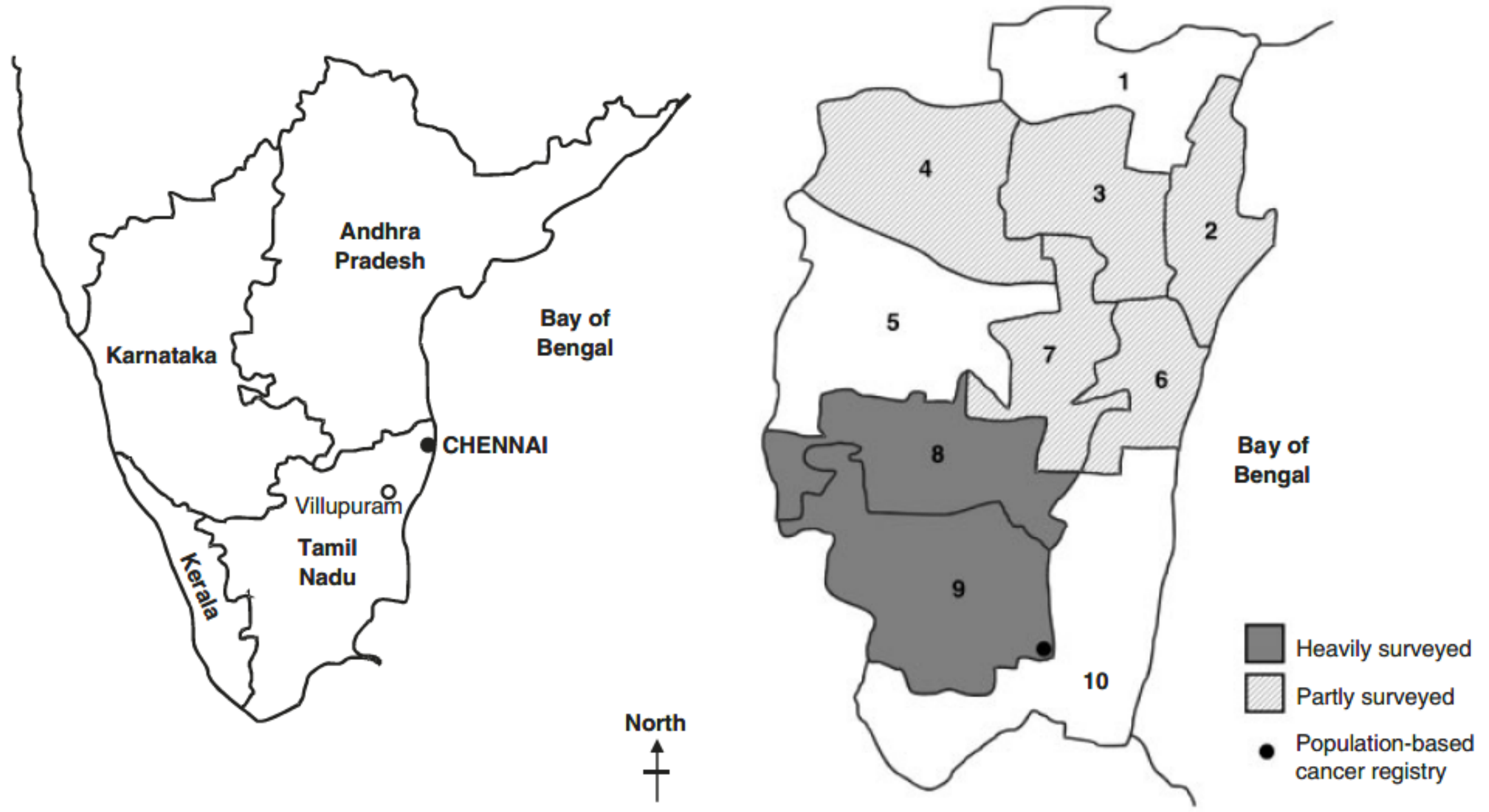

*Excluding urban areas outside the City Corporation boundaries and hence outside the study area

English translation of the Tamil-language baseline survey form used in 1998–2001 by fieldworkers visiting all households in randomly chosen streets in Chennai city

City zone (1-10) ☐ ☐ Project ID No. ☐☐☐☐☐

Division No. ☐ ☐ ☐ Result of visit (1-4, from Part 4) ☐

Part 1 - Identification details for the entire household

1. **Full name of the head of the household:**
   and if male, father's name and if female, spouse's name:
2. **Name of the respondent:**
   and relationship to head, if different:
3. **Ration card holder (Y/N)**
   and if Yes, ration card number:
4. **Present residential address:**
   and second address in Chennai, if any:
5. **Religion:**

6. **Mother tongue:**

7. **Profile of all persons in the household** [add extra lines if more than 10 are needed]

	Name	Age	Sex	Marital status*	Years of education	Relation to head	Any habits?** If Yes, list all	Occu-pation
1								
2								
3								
4								
5								
6								
7								
8								
9								
10								

* U=Unmarried, M=Married, W=Widowed, D=Divorced, S=Separated

** SM=Smoking, CH=Chewing areca/betel/tobacco quids, SF=Snuff use, AL=Alcohol drinking

8. **Socio-economic status of the household**

☐ Type of house (1 Individual, 2 Flat, 3 Portion, 4 Hut, 5. Other)
☐ Number of rooms
☐ Separate bathroom (0 No, 1 Yes)
☐ Fuel used for cooking (1 Gas, 2 Wood, 3 Kerosene)
☐ Vehicle (0 None, 1 Car, 2 Two-wheeler, 3 Cycle, 4 Other: specify)
☐ Refrigerator (0 No, 1 Yes)
☐ House (0 Owner, 1 Tenant)
☐ Television (0 No, 1 Only black and white, 2 Colour TV)
☐ Wet grinder (0 No, 1 Yes)

9. **Any cancer in the family (Y/N)? [If more than one, give this information for each case]**

Name: Sex: Relationship to head: Age at onset:

Year cancer diagnosed: Site of cancer origin (if known): Alive/dead?

Part 2 – MEN. Questions to be asked only to men of age 35 or older

1. **Name:** **Father's name:** **Wife's name:**
2. **Age (years):** **Date of birth:** **Place of birth:**
3. **Height (cm):** **Weight (kg):** **Waist (cm):**
4. **FIRST blood pressure reading (SBP/ DBP, mmHg): ……../ ……..**
5. **Peak expiratory flow rate (L / minute):**
6. **Main occupations**

	Occupation	Main employer	Describe any significant chemical exposure at work	Age began	Age stopped
1					
2					

7. **Diet:** ☐Vegetarian, no eggs/ ☐Non-vegetarian/ ☐Vegetarian with eggs; Brahmin diet? (Y/N)
8. **Previous history of treated disease**

	Y/N	Time since onset		Y/N	Time since onset
Hypertension			Cancer (& site)		
Asthma			Stroke/paralysis		
Diabetes			Heart attack		
Tuberculosis			Other (specify*)		

*Coded later as cardiac/not

9. **Habits – smoking, chewing, snuff-taking, drinking**

Smoking	0/blank=never, 1=ex, 2=current	Frequency per day	Age began	Age stopped	Duration (years)
Bidis					
Cigarettes					
Other (specify)					
Circle places you smoke: 1. House, 2. Toilet, 3. Office, 4. Bus Stop, 5. Rly Stn, 6. Street, 7. Other<br>Time spent per day smoking (minutes): Money spent per month on smoking (Rs):					

Quid chewing or snuff-taking	0/blank=never, 1=ex, 2=current	Frequency per day	Age began	Age stopped	Duration (years)
Areca nut (AN) alone					
Tobacco alone					
AN + betel leaf (BL)					
AN + tobacco					
AN + BL + tobacco					
Manufactured quid<br>- containing tobacco					
- without tobacco					
**Snuff-taking**					

Alcohol drinking	0/blank=never, 1=ex, 2=current	Frequency per week	Amount /event	Age began	Age stopped	Duration (years)
Indian liquor (arrack/toddy)						
Whisky						
Beer						
Brandy						
Other (specify)						

10. **SECOND blood pressure reading (SBP/DBP, mmHg): ……../ ……..** **[Jump to Part 4]**

Part 3 – WOMEN. Questions to be asked only to women of age 35 or older

1. **Name:** **Husband's name:**
2. **Age (years):** **Date of birth:** **Place of birth:**
3. **Height (cm):** **Weight (kg):**
4. **FIRST blood pressure reading (SBP/ DBP, mmHg): ……../ ……..**
5. **Peak expiratory flow rate (L / minute):**
6. **Main occupations**

	Occupation	Main employer	Describe any significant chemical exposure at work	Age began	Age stopped
1					
2					

7. **Diet:** ☐Vegetarian, no eggs/ ☐Non-vegetarian/ ☐Vegetarian with eggs; Brahmin diet? (Y/N)
8. **Previous history of treated disease**

	Y/N	Time since onset		Y/N	Time since onset
Hypertension			Cancer (& site)		
Asthma			Stroke/paralysis		
Diabetes			Heart attack		
Tuberculosis			Other (specify*)		

*Coded later as cardiac/not

9. **Habits – smoking, chewing, snuff-taking, drinking**

	0/blank=never, 1=ex, 2=current	Frequency per day	Age began	Age stopped	Duration (years)
**Smoking:** Bidis					
Cigarettes					
Other (specify)					
**Chewing:** AN alone					
Tobacco alone					
AN + betel leaf (BL)					
AN + tobacco					
AN + BL + tobacco					
Manufactured quid - containing tobacco					
- without tobacco					
**Snuff-taking**					
**Alcohol** (specify type, frequency & quantity)					

10. **SECOND blood pressure reading (SBP/DBP, mmHg): ……../ ……..**

Part 4: Final result of visit(s) to this household

☐ 1=Complete information, 2=Partial information, 3=No adult ever available / house locked, etc
4=Interview refused – give reason(s):

☐ No. of visits undertaken, and date of visit 1: of visit 2: of visit 3:

Interviewed by: **Date of final interview:**

Supplementary Table 1: Categorisation of ICD-10 codes for death causes, and category-specific numbers of deaths

Endpoint name	ICD-10 codes	Number of deaths, 35-69 y	
		Men	Women
VASCULAR			
Cardiac	I20-I25	12 526	7 472
Stroke	I60-I64	1 674	1 585
RENAL	E10.2, E11.2, E12.2, E13.2, E14.2, N00-N29	2 153	2 131
CANCER	C00-C97	3 274	3 556
RESPIRATORY			
Respiratory TB	A15-A16	2 909	466
Other respiratory	J00-J99	1 186	971
Non-medical	V01-Y98	2 495	1 142
OTHER OR UNSPECIFIED MEDICAL*	Any ICD-10 code excluding the cause-specific mortality codes listed above	5 120	3 467
All medical	Any ICD-10 code excluding V01-Y98	28 842	19 648
ALL CAUSES	A00-Y98	31 337	20 790

*Other or unspecified medical causes of death: 852 deaths from other infections, 28 deaths from haematologic disorders, 1686 deaths from diabetes (excluding deaths from diabetes with renal complications), 26 deaths from mental disorders, 241 deaths from nervous system disorders, 390 deaths from unspecified heart disease, 1671 deaths from digestive system disorders, 4 deaths from obstetric disorders and 3689 deaths from unspecified causes.

Supplementary Table 2: Numbers of participants recruited and numbers excluded, by reason for exclusion and by sex

	Men	Women	All
**Initial number of participants**	264,846	235,964	500,810
**Excluded due to:**			
Age ≥70 at baseline	16,796	16,527	33,323
Asthma	2,461	2,621	5,082
TB	1,115	405	1,520
Heart attack	732	411	1,143
Stroke	578	284	862
Cancer	72	199	271
**Total in main analyses**	**243,092**	**215,517**	**458,609**

Supplementary Table 3: Age dependence of baseline characteristics, by sex

(a) Men

	Years of education				
	**None**	**1-7**	**8-11**	**>11**	**Overall**
**N**	23,385	78,595	98,424	42,688	243,092
**Age (years)**	47.8 (10.0)	46.3 (9.6)	45.4 (8.9)	45.1 (8.5)	45.9 (9.2)
**Current tobacco smoker**	12,396 (53%)	36,047 (46%)	30,382 (31%)	6,782 (16%)	85,607 (35%)
**- Total of cigarettes or bidi per day**	13 (8)	12 (7)	11 (6)	10 (6)	11 (7)
**Only cigarettes**					
**- N (% of current)**	5,399 (44%)	22,171 (62%)	24,342 (80%)	6,360 (94%)	58,272 (68%)
**- Cigarettes/day**	10 (5)	10 (5)	10 (5)	9 (5)	10 (5)
**Only bidis**					
**- N (% of current)**	5,781 (47%)	11,040 (31%)	4,646 (15%)	300 (4%)	21,767 (25%)
**- Bidi/day**	15 (8)	15 (8)	14 (7)	13 (7)	14 (8)
**Remaining current smokers**	1,216 (10%)	2,836 (8%)	1,394 (5%)	122 (2%)	5,568 (7%)
**- Total of cigarettes or bidi per day**	19 (11)	19 (11)	18 (11)	16 (13)	19 (11)
**Ex-smokers, N (%)**	507 (2%)	1,886 (2%)	1,599 (2%)	572 (1%)	4,564 (2%)
**Ever tobacco smoker**	12,903 (55%)	37,933 (48%)	31,981 (33%)	7,354 (17%)	90,171 (37%)
**- Total of cigarettes or bidi per day when last smoked**	13 (8)	12 (7)	11 (6)	10 (6)	11 (7)
**- Age at starting**	21.2 (4.2)	21.7 (4.2)	22.3 (4.1)	23.0 (4.1)	21.9 (4.2)
**Current alcohol drinker**	10,506 (45%)	27,418 (35%)	21,682 (22%)	4,206 (10%)	63,812 (26%)
**- Alcohol/week (g)***	211 (431)	193 (440)	172 (429)	160 (497)	187 (439)
**Ever drank alcohol**	10,951 (47%)	28,833 (37%)	22,784 (23%)	4,482 (11%)	67,050 (28%)
**- Alcohol/week (g) when last drank**	215 (458)	195 (446)	174 (429)	164 (566)	189 (452)
**Ex-alcohol drinker, N (%)**	445 (2%)	1,415 (2%)	1,102 (1%)	276 (1%)	3,238 (1%)
**Ever smoked or drank**	15,145 (65%)	44,001 (56%)	38,176 (39%)	8,828 (21%)	106,150 (44%)
**Ever smoked, drank or chewed**	16,130 (69%)	46,326 (59%)	40,679 (41%)	9,581 (22%)	112,716 (46%)
**Vegetarian**	184 (1%)	1,014 (1%)	4,329 (4%)	4,555 (11%)	10,082 (4%)
**Current quid chewing†**	2,689 (12%)	5,582 (7%)	4,681 (5%)	1,145 (3%)	14,097 (6%)
**Lowest SES group**	11,694 (50%)	29,337 (37%)	20,131 (21%)	2,579 (6%)	63,741 (26%)
**BMI (kg/m²)**	21.4 (3.2)	22.1 (3.3)	22.9 (3.2)	23.6 (3.2)	22.6 (3.3)
**BMI <20**	7,017 (30%)	17,857 (23%)	14,895 (15%)	3,646 (9%)	43,415 (18%)
**Self-reported diabetes**	558 (2%)	3,257 (4%)	5,094 (5%)	2,513 (6%)	11,422 (5%)
**SBP**	127 (15)	127 (14)	128 (14)	128 (13)	128 (14)
**DBP**	82 (9)	83 (9)	84 (9)	84 (8)	83 (9)
**Hypertension**	6,206 (28%)	22,022 (29%)	29,371 (31%)	12,944 (33%)	70,543 (31%)
**Treated, N (% of hypertension)**	429 (7%)	2,276 (10%)	3,688 (13%)	1,923 (15%)	8,316 (12%)

(b) Women

	Years of education				
	**None**	**1-7**	**8-11**	**>11**	**Overall**
**N**	62,907	81,616	57,239	13,755	215,517
**Age (years)**	49.7 (9.8)	46.5 (9.3)	44.5 (8.3)	43.4 (7.5)	46.7 (9.4)
**Ever tobacco smoker**	52 (0.1%)	38 (0.0%)	7 (0.0%)	3 (0.0%)	100 (0.0%)
**Ever drank alcohol**	62 (0.1%)	31 (0.0%)	17 (0.0%)	2 (0.0%)	112 (0.1%)
**Ever smoked or drank**	104 (0.2%)	60 (0.1%)	23 (0.0%)	4 (0.0%)	191 (0.1%)
**Vegetarian**	3,204 (5%)	5,982 (7%)	7,279 (13%)	2,482 (18%)	18,947 (9%)
**Current quid chewing†**	13,372 (21%)	5,626 (7%)	1,568 (3%)	184 (1%)	20,750 (10%)
**Lowest SES group**	27,132 (43%)	22,127 (27%)	7,816 (14%)	619 (5%)	57,694 (27%)
**BMI (kg/m^2)**	23.1 (4.4)	24.2 (4.5)	24.6 (4.3)	24.8 (4.0)	24.0 (4.4)
**BMI <20**	13,136 (21%)	11,657 (14%)	6,329 (11%)	1,016 (7%)	32,138 (15%)
**Self-reported diabetes**	3,296 (5%)	4,729 (6%)	2,683 (5%)	502 (4%)	11,210 (5%)
**SBP**	128 (18)	127 (18)	126 (16)	125 (15)	127 (17)
**DBP**	83 (11)	83 (10)	82 (10)	82 (9)	83 (10)
**Hypertension**	19,295 (34%)	24,291 (33%)	14,767 (30%)	2,936 (26%)	61,289 (32%)
**Treated, N (% of hypertension)**	3,057 (16%)	4,689 (19%)	2,794 (19%)	516 (18%)	11,056 (18%)

Values are % (and number) or mean (SD), as appropriate.
SBP = systolic blood pressure; DBP = diastolic blood pressure; BMI = body mass index; some participants did not contribute values for these variables.
Hypertension = SBP≥140 or DBP≥90 mm Hg or treatment for hypertension (as a surrogate for diagnosis, since this was not directly reported)
*: Past/current intake, assuming alcohol by volume: spirits 40%, wine 12%, beer 4%, toddy 5%.
†: Any quid chewing: tobacco, non-tobacco or combination, whether regular or occasional
SES was a composite household-asset-based score obtained by summing scores based on several self-reported indicators: housing type and number of rooms; housing ownership; type of cooking fuel; and household ownership of a bicycle, motor-cycle, car, refrigerator, television, or electric grain grinder. SES at baseline and at resurvey were calculated using the same rules and the cut-points were quartiles of the baseline score.

Supplementary Table 4: Education and SES at baseline and at resurvey about 14 years later, by sex

A) Years of education, men (Kappa=0.88)

Reported at resurvey	Reported at baseline			
	**None** (n=747)	**1-7** (n=864)	**8-11** (n=4465)	**>11** (n=1008)
**None (n=781)**	671	63	34	13
**1-7 (n=863)**	70	705	76	12
**8-11 (n=4340)**	6	96	4169	69
**>11 (n=1100)**	0	0	186	914

B) SES, men (Kappa=0.18)

Reported at resurvey	Reported at baseline			
	**I** (n=1829)	**II** (n=1873)	**III** (n=1615)	**IV** (n=1810)
**I (n=312)**	224	56	22	10
**II (n=545)**	298	136	83	28
**III (n=1875)**	561	647	443	224
**IV (n=4395)**	746	1034	1067	1548

C) Years of education, women (Kappa=0.91)

Reported at resurvey	Reported at baseline			
	**None** (n=1118)	**1-7** (n=556)	**8-11** (n=1206)	**>11** (n=91)
**None (n=1171)**	1084	53	29	5
**1-7 (n=556)**	34	469	46	7
**8-11 (n=1147)**	0	34	1107	6
**>11 (n=97)**	0	0	24	73

D) SES, women (Kappa=0.13)

Reported at resurvey	Reported at baseline			
	**I** (n=936)	**II** (n=971)	**III** (n=661)	**IV** (n=438)
**I (n=105)**	78	18	7	2
**II (n=260)**	150	76	29	5
**III (n=1077)**	402	421	200	54
**IV (n=1564)**	306	456	425	377

SES score is a simple summation of values from each SES element of the questionnaire. Housing type: Hut=1, portion=2, flat=3, individual=4; Rooms: Point per room (max 4); Separate bathroom: No=0, Yes=1; Fuel used for cooking: Wood=1, kerosene=2, gas=3; Vehicle: None=0, cycle=1, two wheeler=2, other=3, car=4; Refrigerator: No=0, Yes=1; House owner: Tenant=0, owner=1; TV: No=0, black and white=1, colour=2; Grinder: No=0, yes=1.

SES at baseline and at resurvey were calculated using the same rules and the cut-points were the quartiles of the baseline score.

Supplementary Table 5: Smoking and drinking at baseline and at resurvey about 14 years later, by sex

A) Smoking status, men

Reported at resurvey	Reported at baseline		
	Current (n=2410)	Ex- (n=122)	Never (n=4595)
Current (n=2251)	2218	28	5
Ex- (n=278)	182	94	2
Never (n=4598)	10	0	4588

B) Drinking status, men

Reported at resurvey	Reported at baseline		
	Current (n=2066)	Ex- (n=117)	Never (n=4944)
Current (n=1976)	1894	37	45
Ex- (n=242)	161	79	2
Never (n=4909)	11	1	4897

C) Smoking status, women

Reported at resurvey	Reported at baseline		
	Current (n=1)	Ex- (n=0)	Never (n=3005)
Current (n=1)	1	0	0
Ex- (n=0)	0	0	0
Never (n=3005)	0	0	3005

D) Drinking status, women

Reported at resurvey	Reported at baseline		
	Current (n=0)	Ex- (n=0)	Never (n=3006)
Ever (n=0)	0	0	0
Ex- (n=0)	0	0	0
Never (n=3006)	0	0	3006

Supplementary Table 6: Smoking at baseline versus that at resurvey among men aged 35-59 at entry and resurveyed 14 years later

Smoking (in 8 categories) at the baseline survey	Number of resurveyed men aged <60 at baseline	Mean (SD) of estimated cigarettes/day		Mean (SD) of estimated bidis/day		Mean (SD) of estimated [cigarettes/day + bidis/day]	
		At baseline	At resurvey	At baseline	At resurvey	At baseline	At resurvey
Cigarettes only, <10/day	643	5.2 (1.9)	4.8 (4.1)	-	2.6 (6.0)	5.2 (1.9)	7.3 (5.4)
Cigarettes only, 10/day	634	10.0 (0.0)	6.8 (4.4)	-	2.0 (5.1)	10.0 (0.0)	8.8 (4.4)
Cigarettes only, >10/day	249	16.9 (5.5)	9.1 (7.4)	-	2.1 (5.6)	16.9 (5.5)	11.3 (7.8)
**Subtotal: Cigarettes only**	1526	9.1 (4.8)	6.3 (5.1)	-	2.3 (5.6)	9.1 (4.8)	8.6 (5.7)
Bidis only, <10/day	90	-	2.7 (6.2)	5.3 (1.8)	6.3 (6.2)	5.3 (1.8)	9.0 (6.7)
Bidis only, 10/day	131	-	1.5 (3.1)	10.0 (0.0)	9.1 (7.0)	10.0 (0.0)	10.6 (5.6)
Bidis only, >10/day	306	-	1.7 (3.4)	19.6 (5.5)	10.8 (9.0)	19.6 (5.5)	12.6 (7.5)
**Subtotal: Bidis only**	527	-	1.8 (4.0)	14.8 (7.2)	9.6 (8.3)	14.8 (7.2)	11.5 (7.1)
All other ever smokers†	287	7.0 (6.4)	5.3 (6.5)	9.1 (8.0)	6.4 (7.7)	16.1 (10.0)	11.6 (8.3)
**Subtotal: Ever smoked**	2340	6.8 (5.8)	5.2 (5.4)	4.4 (7.7)	4.4 (7.3)	11.2 (6.9)	9.6 (6.5)
Never smoked	4138	-	0.0 (0.3)	0.0 (0.0)	0.0 (0.2)	0.0 (0.0)	0.0 (0.3)

† Includes ex-smokers, men who smoked but did not use any cigarettes or bidis, and men who smoked both cigarettes and bidis

Supplementary Table 7: Drinking at baseline versus that at resurvey among men aged 35-59 at entry and resurveyed 14 years later

Alcohol use (days/week) at the BASELINE survey	Number of resurveyed men aged <60 at baseline	Mean (SD) estimated days/week using alcohol		Mean (SD) estimated grams/week of alcohol	
		At baseline	At resurvey	At baseline	At resurvey
Ex-drinker	99	3.5 (2.7)	3.5 (2.7)	217.4 (247.1)	226.9 (310.6)
<2 days/week	925	0.8 (0.3)	2.4 (2.3)	43.8 (22.9)	144.5 (163.2)
2-6 days/week	549	2.6 (0.8)	3.0 (2.1)	150.0 (72.4)	197.5 (225.8)
>6 days/week	476	7.0 (0.0)	3.7 (2.6)	479.6 (491.9)	272.4 (396.7)
**Subtotal: Ever used alcohol**	2049	2.9 (2.5)	2.9 (2.4)	181.9 (300.4)	192.4 (263.4)
Never use alcohol	4429	0.0 (0.0)	0.04 (0.46)	0.0 (0.0)	2.6 (30.7)

Supplementary Table 8: Men: Effects of sequential adjustment for confounders on the all-cause mortality rate ratios for smoking and/or drinking

	RR	(95% CI)	% reduction in log RR
**Smoker vs never-smoker**			
Adjusted only for age	1.74	(1.70-1.78)	-
Adjusted for age and education	1.52	(1.48-1.55)	24%
Adjusted for age, education and SES	1.51	(1.47-1.54)	26%
Adjusted for age, education, SES and drinking	1.26	(1.22-1.29)	59%
**Drinker vs never-drinker**			
Adjusted only for age	1.95	(1.91-2.00)	-
Adjusted for age and education	1.70	(1.66-1.74)	21%
Adjusted for age, education and SES	1.69	(1.65-1.73)	21%
Adjusted for age, education, SES and smoking	1.52	(1.48-1.56)	38%
**Both vs neither**			
Adjusted only for age	2.25	(2.19-2.30)	-
Adjusted for age and education	1.90	(1.85-1.95)	21%
Adjusted for age, education and SES	1.89	(1.84-1.94)	21%

RR = mortality rate ratio

Supplementary Table 9: Numbers of deaths among men associated with only smoking, only drinking, or both

	Total deaths, T	Mortality RR	Excess deaths, T - T/RR
Neither	13110	1.00	0
Smoke only	5281	1.26	1078
Drink only	2580	1.52	879
Both	10366	1.89	4884
Total	31337		6841
	(100%)		(22%)

Risk of premature death from tobacco smoking, alcohol, and social inequalities in urban India

Extended abstract

The combined associations of social inequalities, smoking, and drinking with adult mortality may differ greatly between different populations.

In 1998-2001, 500,810 urban household members aged ≥35 were recruited from Chennai, India, and followed to 2020 for verbal-autopsy-assessed cause-specific mortality. After excluding recruits with chronic disease or age ≥70, 458,609 remained. Educational level (none, 1-7, 8-11, >11 years) was used as a proxy for social inequality. Age-standardised Cox regression, censored at age 70, yielded mortality rate ratios (RRs) by education, smoking, and drinking.

There were 52,127 deaths before age 70. Among adults who never smoked or drank, educational level was strongly inversely associated with each of the major causes of death and, comparing extremes, those with no schooling had three times the overall mortality rate at ages 35-69 of those with >11 years of education (men: RR=2·76, 95%CI 2·58-2·95; women: RR=2·93, 2·69-3·19). Few (<0·1%) women smoked or drank. Among men with no schooling 65% smoked or drank versus only 21% of those with >11 years of education. On average, smokers had started at age 22 (SD 4) and smoked 11 (SD 7) cigarettes or bidis daily, and drinkers consumed 19 (SD 44) 10g-units of alcohol per week. In analyses fitting male educational level, smoking, and drinking simultaneously, smokers had 1·26 (1·22-1·29) times the mortality of never-smokers and drinkers had 1·52 (1·48-1·56) times the mortality of never-drinkers. Hence, men who smoked and drank had 1·89 (1·84-1·94) times the mortality of those with neither habit.

There are substantial social inequalities in premature death even among people who do not smoke or drink. Among men, these inequalities are exacerbated by tobacco and alcohol. About half the deaths in middle age among men who both smoke and drink are due to the excess risks associated with these habits.
