# Supplementary material for: Combined impact of smoking, alcohol and social inequalities on premature mortality in urban India
Source: Nat Health. 2026 Jun 19;1(10):1082–91. doi: 10.1038/s44360-026-00148-4 (PMC13630575; doi:10.1038/s44360-026-00148-4)
Supplement: Supplementary file 3 [file 44360_2026_148_MOESM3_ESM.pdf]

nature portfolio

Peer Review File

Risk of premature death from tobacco smoking, alcohol, and social inequalities in urban India

Corresponding Author: Professor Sarah Lewington

This file contains all reviewer reports in order by version, followed by all author rebuttals in order by version.

Version 0:

Reviewer comments:

Reviewer #1

(Remarks to the Author)
- The submitted paper presents a highly original and significant study quantifying the separate and joint associations of education, smoking and drinking on mortality outcomes in India. These are among the greatest contributors to morbidity and mortality and are particularly important due to the possibilities regarding prevention.

- The impacts of SES inequality, smoking and drinking vary between populations and this study is the largest and most reliable to date in this setting. It is of high quality and conducted according to local needs, including the appropriate use of verbal autopsy, while also applying world-leading statistical and analytic methods.

- Most SES, smoking and drinking findings are from high income countries. The pattern of smoking and drinking in India is not typical of most high income countries.

- This study is of sufficient power to quantify the independent contributions of social inequalities, smoking and drinking, which is unique, and also highly informative.

- We often downplay and normalise extraordinarily harmful exposures and are preoccupied by novel factors and "cures". At the same time, we lament social inequalities in outcomes and wonder how best to address them. This paper is of great value because it demonstrates that half of the deaths in middle aged men who smoke and drink are due to the excess risks related to these – with clear implications for prevention.

- The evidence on the relation of cause-specific mortality to education is especially interesting as is the presentation of joint associations of education, smoking and drinking to all-cause mortality.

- Great to see resurvey and estimation of usual amount smoked during follow up, 14 years after baseline, to account for regression dilution from random errors, or within-person temporal variability. Extraordinary to have >99% agreement on ever/never. It would be useful to have evidence on the proportion quitting who currently smoked or drank at baseline, since the methods state it was uncommon but I'm not able to locate where this is reported. Have looked at tables S6 and S7 but can't ascertain it from them.

- Useful to have data separated by cigarettes and bidis, since these data are often lacking and the findings as described are useful internationally.
- Readers will appreciate the application of relative risks to population official death statistics to obtain absolute risks.

- It is very informative to see the results regarding ever/never drinking and ever/never smoking to cause specific mortality, as well as the findings regarding usual drinking level and cause-specific outcomes. It is interesting and important to note the lack of a "protective" effect of low alcohol consumption on cardiovascular outcomes, which adds findings from this important context to the growing evidence in this area. I'm curious as to why there was no analyses presented of usual cigarette/bidi consumption and cause-specific mortality (ie like Figure S9 for alcohol)?

Data & methodology: validity of approach, quality of data, quality of presentation
- The focus on premature mortality is appropriate, both from a methods point of view and regarding public health

implications. It is also appropriate to exclude people with chronic disease.

- The statistical methods are exemplary. However, the description could be improved by delineating more clearly the sequence of analyses being presented and which exposures and outcomes they relate to. The statistical methods section relates appropriately to various important statistical challenges and how they were dealt with (e.g. avoiding reverse causality by excluding people with illness at baseline, focus on premature mortality to improve reliability, violation of proportional hazards, floating confidence intervals, regression dilution). However, the various analyses are woven into this, making it challenging to clearly follow which analyses were undertaken.

- Terrific to see the results on SES inequalities in mortality in people who neither smoked nor drank, which gives the reader immediate evidence about the scale of SES inequity independent of these factors. Adjustment would not be sufficient. Also good to see smoking and drinking results stratified by education.

- Appreciate the floating relative risks.

- Excellent to have both relative and absolute risks, as well as the crystal clear statement "About half the deaths in middle age among men who both smoke and drink are due to the excess risks associated with these habits".

- Fascinating to see the rapid changes in household assets and the implications for ascertaining SES and reproducibility of measures.

- The persisting relationship of education to mortality, following adjustment for the other SES measure and the lack of strong independent relevance of that measure is both interesting and important.

- The authors note: "Although previous prospective studies in India have demonstrated associations of mortality with smoking, drinking, and lower educational level or socioeconomic status…". It would be helpful to cite the studies outlined below at this point.

- In the discussion, the only strategy the authors mention to reduce social inequalities is access to healthcare. This could be interpreted as attributing much of the large education related disparities to access to healthcare. It would be of benefit to the reader to know more explicitly why the authors consider this likely to substantively reduce them, and if they have any other suggestions of other factors that might reduce inequalities.

- Table 1 would benefit from greater clarity about what the numbers relate to. Assume % (n) mostly. "Frequencies" will not be clear to most people and this doesn't indicate what the figures in parentheses are for most of the contents of the table.

- S4 and S5: although it is clear that these relate to men and women separately, it is unclear from figure title other ways in which they differ from Figure 2 e.g. is it in the whole population or restricted to people who do not smoke or drink? Also would be good to be consistent with titles e.g. Figure 2 says "Associations of education with cause-specific mortality rate ratios…." While Figures S4 and S5 do not include this level of detail.

- Generally good to avoid term "smokers" and "drinkers".

- Would be good to have on figures and tables clarity about whether findings relate to current tobacco smoking (at baseline) or ever. The methods section notes that the main analyses compare ever- versus never users as cessation (before or after baseline) was uncommon. However, it would be good to have that explicit in all tables, as it reflects the unique context and lack of quitting, which is different from other studies which generally relate outcomes to current smoking/drinking at baseline. For example, Figure 4 mentions current cigarette and bidi use at baseline while other figures mention people who "do and do not smoke".

- Previous work is appropriately cited.

- The abstract, introduction and conclusions are clear and appropriate.

(Remarks on code availability)

Reviewer #2

(Remarks to the Author)
Overall an important study with sufficient power to answer a very relevant public health question.
Language improvement
Avoid unusually large.. cohort..
Avoid drinking to mean alcohol consumption – as it is a colloquial term not scientific.
Methods
Given that smoking alcohol consumption are critical variables, their measurement must be provided in detail in this paper and not just referred to an earlier publication.
Statistical analyses
Please clarify that the exclusion of chronic diseases was at baseline.

Exclusion of people above 69 years that they might be less likely to follow up (please give actual numbers ant not hypothesize)
A sub-sample has been resurveyed, and the results show that except improvement in SES, smoking and alcohol, consumption remain largely unchanged. I am not sure why they have to assume that the mean values of subsample at resurvey will apply to ALL subjects.

Ethics. The appropriateness of ethical clearance received at the start of this cohort to remain valid for follow-up and resurvey needs more clarity, given that the PI has retired since and institutional oversight mechanisms are not clear.
Discussion: While comparison of RR for smoking and mortality across studies is fine, it has to be emphasized that this depends on dose of exposure (amount tobacco/alcohol consumed) and a discussion on the differences need to provide this information.
Tables & figures
Table 1 – better labeling BMI SD); Mean SBP, Mean DBP, BMI (SD) ….
Proportion of those on treatment should ideally among only those with known hypertension.; diabetes should also be reported or known diabetes.
Figure 1. There appears no need for two figures as one line is repeated. Given that there are no scaling sues, all three lines could be shown one figure enables better comparison.

(Remarks on code availability)

Reviewer #3

(Remarks to the Author)
This manuscript describes a cohort study of mortality before age 70 among people in India, with follow-up over 20 years, focussing on the interactions between smoking, drinking, SES, education. My comments are below.

1. I appreciate the authors including the statistical code for this project. However, the statement under "Code availability" says "Analytical coding can be shared on request to the corresponding author" - does this mean the intention is not to make it available (e.g. as a supplementary file)? I would highly suggest the code is made available - for instance, the authors could add the files to Github and link to the repository in their paper. Also, the actual code for the Cox analysis ("Run Cox Analysis System v1.sas") does not seem to be provided. Lastly, the metadata within the code could be better - the header says: "COX analysis system for Kadoorie Last edited: 6 Dec 2011 Corinna Hong" which is a bit confusing.

2. The primary outcome (mortality) was identified through a mix of active and passive follow-up. This would have been a substantial amount of work, given the study size (~500,000). As this is the main outcome, it is important that it is captured accurately and completely. Do the authors have any way of knowing how complete this is? Perhaps including information on how many deaths were identified via manual follow-up vs passive follow-up would be informative. Was the observed death rate in study participants consistent with what would be expected?

3. The methods state that “A composite household-asset-based SES score was obtained by summing scores based on several self-reported indicators” Was this score developed by the researchers, or has this been previously validated? Can they describe in more detail exactly how it was calculated? Also, SES was reported as 4 groups – how were these defined in relation to the SES score?

4. People with chronic disease were excluded. First, a justification for this should be given. Second, the list of conditions included is quite short. For instance, myocardial infarction, but no heart disease more generally. No diabetes. Asthma, but no COPD. Looking at the questionnaire, it does ask about hypertension and diabetes, so it seems odd not to include them.

5. The authors have not stated how they dealt with missing data, particularly for key exposures (smoking, drinking). It seems very unlikely there would be no missing data. The amount of missing data should also be reported.

6. I understand the reason for modifying the baseline exposure based on the results of the resurvey to adjust for regression dilution. However, the people resurveyed after 14 years are those who were alive and agreed to be resurveyed, so more likely to be younger/ healthier. I did look at the Clarke et al (1999) paper which investigated the impact of early deaths on adjustment for regression dilution and concluded that it was minimal – however that was in relation to hypertension and high cholesterol, which arguably are less closely related with short-term mortality than smoking/ drinking. Also, wouldn’t assigning the mean intake at resurvey to all participants lead to everyone having the same value within each category? It’s unclear how this information was used – was it just for plotting?

7. “The relevance of education, smoking and drinking to the probability of a 35-year-old dying before age 70 was assessed by combining the risk factor prevalences and age-adjusted RRs in this study with age- and sex-specific all-cause mortality rates from Chennai official statistics.” Can the authors be more specific how this was done?

8. The outcome was mortality before age 70, while the Abstract states people were censored at age 70, this is not stated in the Methods. I suggest adding this.

9. In Table 1, are the values for smoking/drinking the “usual” consumption values estimated based on the resurvey?

10. Table 1 is a mix of n (%) and mean (SD) – a bit more clarity within the table about which is which would help.

11. Figure 1- It took me a minute to realise that "men who do not smoke or drink" are represented on both panels. Is there a reason to have two separate panels here? Couldn't "men who both smoke and drink" be placed on the first panel?

12. Figure S2 – I don't think the chi-square statistic is necessary to include in this figure.

(Remarks on code availability)
I reviewed most of the code (there is a lot) - it seems like most of the files are just templates for rerunning different analyses with slightly different parameters.

I don't think it would be reproducible though as the code that actually runs the Cox regression (i.e. "Run Cox Analysis System v1.sas") does not seem to be included. As mentioned in my review the metadata is confusing as it suggests that the code was written for the Kadoorie study in 2011.

Version 1:

Reviewer comments:

Reviewer #1

(Remarks to the Author)
The authors have addressed all the concerns raised by the reviewers and I have no further comments on it. It is an excellent paper and provides evidence relevant to the LMIC setting, which is very valuable.

(Remarks on code availability)

Reviewer #2

(Remarks to the Author)
The authors have addressed all queries satisfactorily

(Remarks on code availability)

Reviewer #3

(Remarks to the Author)
I am satisfied with the authors' response to my comments, I have nothing further to add.

(Remarks on code availability)
I checked that the code was in fact shared, but I have not checked the actual code itself (as there is a lot).

Open Access This Peer Review File is licensed under a Creative Commons Attribution 4.0 International License, which permits use, sharing, adaptation, distribution and reproduction in any medium or format, as long as you give appropriate credit to the original author(s) and the source, provide a link to the Creative Commons license, and indicate if changes were made.
In cases where reviewers are anonymous, credit should be given to 'Anonymous Referee' and the source.
The images or other third party material in this Peer Review File are included in the article's Creative Commons license, unless indicated otherwise in a credit line to the material. If material is not included in the article's Creative Commons license and your intended use is not permitted by statutory regulation or exceeds the permitted use, you will need to obtain permission directly from the copyright holder.
To view a copy of this license, visit https://creativecommons.org/licenses/by/4.0/

18th February 2026

Dear Dr Soundararajan,

Re: Social inequalities, smoking, drinking, and premature death in India: 20-year prospective study of 500,000 adults

Thank you for your email of 21 October regarding the above manuscript. We have now revised the manuscript in light of the helpful comments from the Editor and reviewers, with responses to the comments detailed in the attached document. We hope that we have addressed all the issues raised satisfactorily and that the manuscript will now be acceptable for publication.

Yours sincerely,

Sarah L. Lewington

Professor Sarah Lewington
Professor of Medical Statistics and Epidemiology

Richard Doll Building, Old Road Campus, Roosevelt Drive, Oxford OX3 7LF
+44(0)1865 743743 enquiries@ndph.ox.ac.uk

Head of Department
Professor Prabhat Jha OC, FRSC

Response to reviewers' comments

(Manuscript NATHEALTH-A01119-T)

REVIEWER #1

1. **Great to see resurvey and estimation of usual amount smoked during follow up, 14 years after baseline, to account for regression dilution from random errors, or within-person temporal variability. Extraordinary to have >99% agreement on ever/never. It would be useful to have evidence on the proportion quitting who currently smoked or drank at baseline, since the methods state it was uncommon but I'm not able to locate where this is reported. Have looked at tables S6 and S7 but can't ascertain it from them.**

   Response – **DONE**. We have now split out Ex- from Current smokers and drinkers in Supplementary Table 5, and the following text has been added, "*Among men, at re-survey, 7.5% (182/2410) of those who reported current smoking at baseline had quit, and 7.8% (161/2066) of those who reported current drinking at baseline had quit. Hence, 97% (6900/7127) reported the same smoking status at baseline and re-survey and 96% (6870/7127) reported the same drinking status at baseline and re-survey*" (lines 199-203).

2. **It is very informative to see the results regarding ever/never drinking and ever/never smoking to cause specific mortality, as well as the findings regarding usual drinking level and cause-specific outcomes. It is interesting and important to note the lack of a "protective" effect of low alcohol consumption on cardiovascular outcomes, which adds findings from this important context to the growing evidence in this area. I'm curious as to why there was no analyses presented of usual cigarette/bidi consumption and cause-specific mortality (ie like Figure S9 for alcohol)?**

   Response – **DONE**. This has now been added to the supplementary appendix (**Extended Data Figures S7 and S8**, line 274). NB, Old Figures S8 and S9 have been re-numbered to **Extended Data Figures S9 and S10**.

3. **The statistical methods are exemplary. However, the description could be improved by delineating more clearly the sequence of analyses being presented and which exposures and outcomes they relate to. The statistical methods section relates appropriately to various important statistical challenges and how they were dealt with (e.g. avoiding reverse causality by excluding people with illness at baseline, focus on premature mortality to improve reliability, violation of proportional hazards, floating confidence intervals, regression dilution). However, the various analyses are woven into this, making it challenging to clearly follow which analyses were undertaken.**

   Response – **DONE**. The 'Statistical analyses' section has been re-structured (lines 102-168) so that it introduces the Cox model, followed by variance of the log risk (floating CIs), regression dilution, and avoiding reverse-causality. We hope this is a better flow.

4. **The authors note: "Although previous prospective studies in India have demonstrated associations of mortality with smoking, drinking, and lower educational level or socioeconomic status…". It would be helpful to cite the studies outlined below at this point.**

   Response – **DONE**. We have added the citations as suggested (line 301).

5. **In the discussion, the only strategy the authors mention to reduce social inequalities is access to healthcare. This could be interpreted as attributing much of the large education related disparities to access to healthcare. It would be of benefit to the reader to know more explicitly why the authors consider this likely to substantively reduce them, and if they have any other suggestions of other factors that might reduce inequalities.**

   Response – DONE. We have slightly edited the text to indicate that the inverse associations of education with many causes of death might be reflecting "disparities in access to effective healthcare" (line 359), but our data do not allow us to explore or quantify these disparities. We have also added that inequalities in health might also be reduced by addressing other wider determinants of health (eg, housing, lifestyle factors) (lines 365-368).

6. **Table 1 would benefit from greater clarity about what the numbers relate to. Assume % (n) mostly. "Frequencies" will not be clear to most people and this doesn't indicate what the figures in parentheses are for most of the contents of the table**.

   Response – **DONE**. We have changed "Frequencies" to "N(%)" and changed the table layout to reflect this. We have also clarified where percentages are of the whole study population or among a specific subset (eg the % of cigarette only among current tobacco smokers).

7. **Figures S4 and S5: although it is clear that these relate to men and women separately, it is unclear from figure title other ways in which they differ from Figure 2 e.g. is it in the whole population or restricted to people who do not smoke or drink? Also would be good to be consistent with titles e.g. Figure 2 says "Associations of education with cause-specific mortality rate ratios…." While Figures S4 and S5 do not include this level of detail**.

   Response – **DONE**. The figure titles have been edited to be consistent with Figure 2.

8. **Generally good to avoid term "smokers" and "drinkers".**

   Response – **DONE**. Changed to men/women who smoked/drank. We have retained "drinker" and "smoker" in some cases where we felt it became quite clumsy but are happy to be advised. For example, we have retained "bidi smoker" and "cigarette smoker" in line 261.

9. **Would be good to have on figures and tables clarity about whether findings relate to current tobacco smoking (at baseline) or ever. The methods section notes that the main analyses compare ever- versus never users as cessation (before or after baseline) was uncommon. However, it would be good to have that explicit in all tables, as it reflects the unique context and lack of quitting, which is different from other studies which generally relate outcomes to current smoking/drinking at baseline. For example, Figure 4 mentions current cigarette and bidi use at baseline while other figures mention people who "do and do not smoke".**

   Response – **DONE**. We have edited the text, and made it explicit in titles and footnotes of figures and tables.

REVIEWER #2

1. **Language improvement. Avoid unusually large.. cohort.**

Response – **DONE**. We have replaced with "*the largest prospective study in India – involving 500,000 urban men and women aged ≥35 years in Chennai city – of the independent and joint associations of education, adult SES, smoking, and alcohol drinking with mortality before age 70.*" (lines 40-43).

2. **Avoid drinking to mean alcohol consumption – as it is a colloquial term not scientific.**

   Response – **DONE**. We have clarified in the title and elsewhere that we studied tobacco smoking and alcohol drinking. As also advised by reviewer 1, we have mostly replaced "smoker" and "drinker" with "men who smoked" and "men who drank alcohol".

3. **Methods: Given that smoking and alcohol consumption are critical variables, their measurement must be provided in detail in this paper and not just referred to an earlier publication.**

   Response – **DONE**. All data were self-reported via questionnaires administered by trained interviewers. This has now been stated explicitly (lines 74-79).

4. **Please clarify that the exclusion of chronic diseases was at baseline.**

   Response – **DONE**.

5. **Exclusion of people above 69 years that they might be less likely to follow up (please give actual numbers and not hypothesize).**

   Response – **DONE**. 7651 (24%) of participants who were aged 70+ at recruitment and were not known to be dead by 1 January 2020 were no longer in active follow-up. However, the main reason for restricting to deaths in middle-age (ie <70 years) is due to the much higher proportion of deaths of unknown cause among those deaths happening at age 70 or above. The following text has been added: "*and are of mortality at ages 35-69, since verbal autopsy arrives at the probable underlying cause of death reliably in middle-age (7% of deaths before 70 were from unspecified causes in this study; Supplementary Table 1) but less so at older ages (42% of deaths at 70 or older were from unspecified causes)*" (lines 105-110)

6. **A sub-sample has been resurveyed, and the results show that except improvement in SES, smoking and alcohol, consumption remain largely unchanged. I am not sure why they have to assume that the mean values of subsample at resurvey will apply to ALL subjects.**

   Response – **DONE**. The following tables (using values from Table 1 and Supplementary Tables 6 and 7) compare baseline consumption among all men to those re-surveyed, showing that the sample re-surveyed are representative of all men. We have added a reference to these tables to the text (lines 205-210).

Among men who were current smokers at baseline

	Values **at baseline** among:	
	All men	Re-surveyed men
Cigarettes only, N	58,272	1,526
Cigarettes/day	10 (5)	9 (5)
Bidi only, N	21,767	527
Bidi/day	14 (8)	15 (7)
Cigarettes + bidi, N	85,607	2340

Cigs+bidi/day	11 (7)	11 (7)

Among men who currently consumed alcohol at baseline

	All men	Re-surveyed men
N	63,812	2049
Days/week	2.7 (2.5)	2.9 (2.5)
g/week	187 (439)	182 (300)

7. **Ethics. The appropriateness of ethical clearance received at the start of this cohort to remain valid for follow-up and resurvey needs more clarity, given that the PI has retired since and institutional oversight mechanisms are not clear.**

   Response – **DONE**. The PI is still active and is Director of the Epidemiological Research Center, Chennai. The Center's Institutional Ethics Committee now has ethical oversight for the study. We have removed "(where the principal investigator, VG, was working)" to avoid confusion (line 172).

8. **Discussion: While comparison of RR for smoking and mortality across studies is fine, it has to be emphasized that this depends on dose of exposure (amount tobacco/alcohol consumed) and a discussion on the differences need to provide this information.**

   Response – **DONE.** We have now acknowledged these important caveats: "*However, these comparisons are dependent upon the quantity smoked (which differed between studies) and duration (which was not consistently reported).*" (line 311-313).

9. **Table 1 – better labelling BMI SD); Mean SBP, Mean DBP, BMI (SD) ….**

   Response – **DONE**. See response to Reviewer 1's sixth comment.

10. **Proportion of those on treatment should ideally be among only those with known hypertension.; diabetes should also be reported or known diabetes.**
    Response – **DONE**. We have added % with measured hypertension (SBP≥140 mmHg or DBP≥90 mmHg) and % of these treated. We have made clearer that diabetes is self-reported.

11. **Figure 1. There appears no need for two figures as one line is repeated. Given that there are no scaling issues, all three lines could be shown one figure enables better comparison.**

    Response – NOT DONE. We prefer to leave this as 2 panels to emphasise the effect of education separately in men and women among never smokers or drinkers and then the added effect of smoking and drinking among men. We will, however, combine them if the editor prefers.

REVIEWER #3

1. **I appreciate the authors including the statistical code for this project. However, the statement under "Code availability" says "Analytical coding can be shared on request to the corresponding author" - does this mean the intention is not to make it available (e.g. as a supplementary file)? I would highly suggest the code is made available - for instance, the authors could add the files to Github and link to the**

repository in their paper. Also, the actual code for the Cox analysis ("Run Cox Analysis System v1.sas") does not seem to be provided. Lastly, the metadata within the code could be better - the header says: "COX analysis system for Kadoorie Last edited: 6 Dec 2011 Corinna Hong" which is a bit confusing.

Response – **DONE**. The analyses used an in-house analysis system which was developed to run age-specific Cox analyses (Prospective Studies Collaboration, *Lancet* 2002; **360**: 1903–13). This in-house system has been transferred to GitHub (https://github.com/PaulSherliker/Global-Population-Studies/tree/Cox-Macro) with a brief readme file (*READme.md*). As an example, the calling programme (*synthetic data analysis - figure 2.sas*) and a dummy dataset, (*synth.sas7bdat*) are in https://github.com/PaulSherliker/Global-Population-Studies/tree/Chennai-Study. This programme will create a PDF of the output that would be used to create Figure 2. A PDF of the actual output used in Figure 2 from running the programme on real data is included in the resubmission. The informed consent does not allow public sharing of the data.

2. **The primary outcome (mortality) was identified through a mix of active and passive follow-up. This would have been a substantial amount of work, given the study size (~500,000). As this is the main outcome, it is important that it is captured accurately and completely. Do the authors have any way of knowing how complete this is? Perhaps including information on how many deaths were identified via manual follow-up vs passive follow-up would be informative. Was the observed death rate in study participants consistent with what would be expected?**

   Response – **DONE**. Although deaths can be identified through the state mortality register, active follow-up was necessary in order to complete verbal autopsies to determine the cause of death. Passive follow-up alone was used only for participants who could not be contacted to determine fact of death. 47436 (91%) were identified by active follow- up and 4691(9%) 6409 were identified through passive follow-up alone. The observed death rates are comparable to mortality rates for Chennai in 2010 (the mean year of death in the study) as shown in the table below.

Age at risk	% of expected
35-79	105%
35-69	105%
**45-69**	**108%**

3. **The methods state that "A composite household-asset-based SES score was obtained by summing scores based on several self-reported indicators" Was this score developed by the researchers, or has this been previously validated? Can they describe in more detail exactly how it was calculated? Also, SES was reported as 4 groups – how were these defined in relation to the SES score?**

   Response – **DONE**. The score was a composite household-asset-based SES score "obtained by summing scores based on several self-reported indicators: housing type and number of rooms; housing ownership; type of cooking fuel; and household ownership of a bicycle, motor-cycle, car, refrigerator, television, or electric grain grinder (but not individual characteristics, eg, caste, religion, occupation, or income)." The possible scores allocated to each item are shown in the footnote to **Supplementary Table 4**. Participants were then divided into 4 equal sized groups with the 25%, 50% and 75% percentiles used for cut-offs. We have added, "*and the score grouped into quartiles.*" (line 74)

4. **People with chronic disease were excluded. First, a justification for this should be given. Second, the list of conditions included is quite short. For instance, myocardial infarction, but no heart disease more generally. No diabetes. Asthma, but no COPD. Looking at the questionnaire, it does ask about hypertension and diabetes, so it seems odd not to include them.**

   Response – PARTIALLY DONE. The main analyses excluded participants with major chronic disease "*to avoid or limit reverse causality*" (now clarified in the text, line 104). The questionnaire only asked specifically about myocardial infarction ("heart attack"), stroke ("paralysis/stroke"), cancer, asthma, tuberculosis, diabetes and hypertension. We wanted only to exclude participants if it made a material difference to the results, and since the prevalence of self-reported diabetes and self-reported hypertension were only 5% and 4%, respectively, at baseline (now added to Table 1), exclusion of these participants did not alter the findings.

5. **The authors have not stated how they dealt with missing data, particularly for key exposures (smoking, drinking). It seems very unlikely there would be no missing data. The amount of missing data should also be reported.**

   Response –**Supplementary Table 2** provides, overall and by sex, the number of participants recruited and the numbers excluded by reason for exclusion (age and prior diseases). There were no missing data among the included 458,609 participants since data were collected by fieldworker-administered questionnaire and participants were willing to answer all questions and have BP, height and weight measured.

6. **I understand the reason for modifying the baseline exposure based on the results of the resurvey to adjust for regression dilution. However, the people resurveyed after 14 years are those who were alive and agreed to be resurveyed, so more likely to be younger/ healthier. I did look at the Clarke et al (1999) paper which investigated the impact of early deaths on adjustment for regression dilution and concluded that it was minimal – however that was in relation to hypertension and high cholesterol, which arguably are less closely related with short-term mortality than smoking/ drinking. Also, wouldn't assigning the mean intake at resurvey to all participants lead to everyone having the same value within each category? It's unclear how this information was used – was it just for plotting?**

   Response – The mean age at baseline among all participants was 46.3 years and was only slightly lower at 44.9 years among those resurveyed. The percentage self-reporting diabetes and hypertension were, respectively, 4.9% (22,632/458,609) and 4.2% (19,372/458,609) at baseline and 4.4% (448/10,133) and 3.5% (355/10,133) at re-survey.

   We apologise if it is not clear how the mean intake at re-survey was used. We did not re-assign values to any individual participant. Rather we divided participants by their self-reported habits at baseline and calculated RRs for each of these baseline-defined groups, and plotted those RRs against the mean at re-survey in the baseline-defined groups. We have clarified this in the text (lines 140-153).

7. **"The relevance of education, smoking and drinking to the probability of a 35-year-old dying before age 70 was assessed by combining the risk factor prevalences and age-adjusted RRs in this study with age- and sex-specific all-cause mortality rates from Chennai official statistics." Can the authors be more specific how this was done?**

   Response – **DONE**. The following text has been added: "*The probability of a 35-year-old dying before age 70 was calculated by multiplying the RRs by a common constant to make their inverse-variance-weighted average match the uniformly age-standardised*

death rate per 1000 person-years at ages 35–69 years (ie, the simple mean of the seven age-specific rates at ages 35–39 years to 65–69 years) in urban Tamil Nadu in 2010 (the median year of death in the study)." (lines 162-167). Details of the method are given in Peto R, Watt J, Boreham J. Deaths from smoking. 2006. http://www.deathsfromsmoking.net/ and this reference has also been added.

8. **The outcome was mortality before age 70, while the Abstract states people were censored at age 70, this is not stated in the Methods. I suggest adding this.**

   Response – **DONE**. This has been added to the Statistical analyses (line 114).

9. **In Table 1, are the values for smoking/drinking the "usual" consumption values estimated based on the resurvey?**

   Response – Table 1 describes the baseline characteristics of the cohort, so these values are the means at baseline.

10. **Table 1 is a mix of n (%) and mean (SD) – a bit more clarity within the table about which is which would help.**

    Response – **DONE**. See response to Reviewer 1's sixth comment.

11. **Figure 1- It took me a minute to realise that "men who do not smoke or drink" are represented on both panels. Is there a reason to have two separate panels here? Couldn't "men who both smoke and drink" be placed on the first panel?**

    Response – See response to Reviewer 2's eleventh comment.

12. **Figure S2 – I don't think the chi-square statistic is necessary to include in this figure.**

    Response – The $\chi 2$ statistic is the change in the model $\chi 2$ on addition of education or SES, respectively, and is provided to show the relative informativeness of Education and SES. We would like to keep this statistic on the figure, and have added a footnote to explain this.

13. **Remarks on code availability: I reviewed most of the code (there is a lot) - it seems like most of the files are just templates for rerunning different analyses with slightly different parameters. I don't think it would be reproducible though as the code that actually runs the Cox regression (i.e. "Run Cox Analysis System v1.sas") does not seem to be included. As mentioned in my review the metadata is confusing as it suggests that the code was written for the Kadoorie study in 2011**.

    Response – **DONE**. The code and dummy data from Figure 2 are now on GitHub (see more detailed response to comment 1).

Response to reviewers' comments (8th April 2026)

(Manuscript NATHEALTH-A01119-T)

We thank the reviewers for their time reviewing and improving this manuscript. Any further changes are in response to the editor's checklist. Page numbers refer to the tracked changes version of the manuscript.

Reviewer #1:
Remarks to the Author:
The authors have addressed all the concerns raised by the reviewers and I have no further comments on it. It is an excellent paper and provides evidence relevant to the LMIC setting, which is very valuable.

Reviewer #2:
Remarks to the Author:
The authors have addressed all queries satisfactorily

Reviewer #3:
Remarks to the Author:
I am satisfied with the authors' response to my comments, I have nothing further to add.

Remarks on code availability:
I checked that the code was in fact shared, but I have not checked the actual code itself (as there is a lot).
